# Valley interference and spin exchange at the atomic scale in silicon

**DOI:** 10.1038/s41467-020-19835-1

**Published:** 2020-11-30

**Authors:** B. Voisin, J. Bocquel, A. Tankasala, M. Usman, J. Salfi, R. Rahman, M. Y. Simmons, L. C. L. Hollenberg, S. Rogge

**Affiliations:** 1 Centre for Quantum Computation and Communication Technology, School of Physics, The University of New South Wales, Sydney, NSW 2052 Australia; 2 Electrical and Computer Engineering Department, Purdue University, West Lafayette, IN USA; 3 Centre for Quantum Computation and Communication Technology, School of Physics, The University of Melbourne, Parkville, Victoria 3010 Australia; 4 School of Computing and Information Systems, Melbourne School of Engineering, The University of Melbourne, Parkville, Victoria 3010 Australia; 5 School of Physics, The University of New South Wales, Sydney, NSW 2052 Australia

**Keywords:** Electronic properties and materials, Information theory and computation, Quantum information

## Abstract

Tunneling is a fundamental quantum process with no classical equivalent, which can compete with Coulomb interactions to give rise to complex phenomena. Phosphorus dopants in silicon can be placed with atomic precision to address the different regimes arising from this competition. However, they exploit wavefunctions relying on crystal band symmetries, which tunneling interactions are inherently sensitive to. Here we directly image lattice-aperiodic valley interference between coupled atoms in silicon using scanning tunneling microscopy. Our atomistic analysis unveils the role of envelope anisotropy, valley interference and dopant placement on the Heisenberg spin exchange interaction. We find that the exchange can become immune to valley interference by engineering in-plane dopant placement along specific crystallographic directions. A vacuum-like behaviour is recovered, where the exchange is maximised to the overlap between the donor orbitals, and pair-to-pair variations limited to a factor of less than 10 considering the accuracy in dopant positioning. This robustness remains over a large range of distances, from the strongly Coulomb interacting regime relevant for high-fidelity quantum computation to strongly coupled donor arrays of interest for quantum simulation in silicon.

## Abstract (web summary)

Coupled donor wavefunctions in silicon are spatially resolved to evidence valley interference processes. An atomic-scale understanding of the interplay between interference, envelope anisotropy and crystal symmetries unveils a placement strategy compatible with existing technology where the exchange is insensitive to interference.

## Introduction

Quantum tunnelling is a widespread phenomena, where a wavefunction leaking as an evanescent mode can be transmitted through a finite barrier. This simple description applies to many natural systems, accounting for instance for molecular conformation or radioactivity^1^. In solid-state systems, nanoscale structures can use tunnelling effects in novel functionalities developed for CMOS electronics^2^ as well as for quantum devices, where electrons can be confined and manipulated in quantum information schemes^3–6^.

For many-body quantum devices, the tunnel interaction *t*, which can couple the different sites, usually competes with the on-site interaction *U* (also called the charging energy). This competition is core to the so-called Fermi-Hubbard model. For fermions, this model needs to be discussed together with the Pauli exclusion principle, which forbids to form a state with two parallel spins on the same orbital^7,8^. When the Coulomb interactions *U* dominate over *t*, the system maps to the Heisenberg model with an exchange interaction which directly links to the tunnel coupling through the relationship *J* =  4*t*^2^/*U*. This Heisenberg limit is favourable for quantum simulations of magnetism^4,9^ and for fast quantum computation schemes^6,10,11^. The mapping to the Heisenberg model breaks down when *t* approaches *U* and when the system is brought away from half-filling, to give rise to a rich phase diagram with links to exotic superconductivity and spin liquids^7,8,12–14^.

**Fig. 1:**
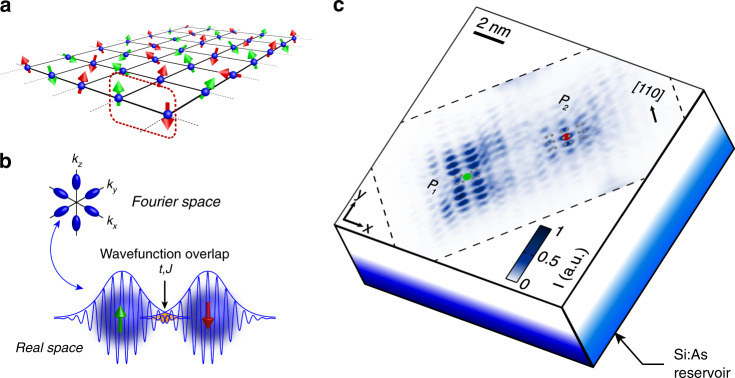
Direct measurement of coupled donors in silicon. **a** Schematics of a 2D-array of single electron spins bound to phosphorus atoms in ^28^Si, where two-qubit operations can occur between nearest neighbours, i.e. red/green spin pairs, using the Heisenberg exchange interaction. **b** (Top) A donor’s electron wavefunction oscillates at the valley wavevector k_*μ*_ ~ 0.81k_0_, with *k*_0_ = 2*π*/*a*_0_. Silicon presents a mass anisotropy which results in the donor orbital envelope to be anisotropic as well, as highlighted by their ovoidal shape in Fourier space. (Bottom) Tunnel and exchange interactions result from the overlap between the two donor orbitals, represented by the yellow overlap area, which is sensitive to both envelope decay and valley interference between donors. **c** Experimental real-space map of the quasi-particle wave function of an exchange-coupled donor pair’s two-electron neutral state. Sequential transport occurs vertically from a highly doped Si:As substrate, which acts as an electron reservoir, to a donor pair found in a low-doped phosphorus *δ*-layer, and then to the STM tip above (not shown). The red and green dots represent the surface projections of the pinpointed lattice positions of the two donors. The two donors are separated by $6.5{a}_{0}\sqrt{2}$ along [110] (perpendicular to the Si dimers, *a*_0_ is the silicon lattice constant), $0.5{a}_{0}\sqrt{2}/2$ (parallel to the Si dimers) along $[1\bar{1}0]$, and by 1.25 *a*_0_ in depth. The grey dots represent the silicon atom positions of the 2 × 1 reconstructed surface. The black dashed contour indicates the experimentally measured area.

Phosphorus donor-bound spins in silicon are highly suitable to explore and harness these different regimes. Donors can be placed at nanometre scale distances from each other in the silicon crystal^15–19^, where direct tunnelling interactions dominate over dipolar coupling. Notably, 2D donor arrays (see Fig. 1a) can be fabricated using scanning tunnelling lithography^5,18^, where the atoms can be placed anywhere in a single atomic plane. These atomically precise devices can be engineered to achieve both the Heisenberg limit^6^ or the non-perturbative tunnelling interactions regime at short inter-dopant distances^7^, with ratios *U*/*t* possibly lower than 10.

The direct relationship between tunnel and exchange coupling can be understood conceptually as they are both based on wavefunction overlap. Hence, they are sensitive to the same physical effects, and these atomic-scale wavefunctions must be precisely investigated to warrant the development of applications which requires the exchange interaction to be well controlled. In contrast to atoms in the vacuum^8^, donor-bound electrons in silicon acquire properties of the crystal band structure. In particular, silicon is an indirect band gap semiconductor^20–22^, with the presence of an anisotropic mass and a valley degree of freedom^23,24^. More precisely, the finite valley momentum is aperiodic with the silicon lattice, a feature which can also be found in 2D material structures^25–27^. This aperiodicity causes interference between oscillating valley states from different donors (see Fig. 1b). Consequently, the tunnel and exchange interactions can vary strongly with small lattice site variations in the donor positions, as they induce changes in both valley interference condition and envelope overlap. Such reduction of the exchange energy compared to hydrogenic systems has been observed in ensemble work^28,29^, but a direct link to valley interference cannot be accessed from any ensemble measurement. Understanding the impact of valley interference at the atomic scale, i.e. at the wavefunction level, has become essential in the context of quantum computing^10,30^, but the predicted valley-induced exchange variations range within five orders of magnitude^10,30–37^, which makes it challenging to design scalable donor qubit architectures with uniform couplings. The sensitivity of both tunnel and exchange interactions to the precise donor positions makes it essential to verify experimentally the presence and magnitude of valley interference. This is challenging to achieve in transport experiments^6,15–19^, which are unable to discriminate between valley interference and envelope effects. Here, we instead implement a real-space probe into coupled donor wavefunctions. This experimental approach leads us to detail the precise role of envelope anisotropy, crystal symmetries, and dopant placement accuracy to reconcile the apparent disparity in the exchange variations predictions. In particular, we consider the dopant placement precision which can be ultimately achieved by scanning probe lithography^5^. We find that exchange variations from pair to pair can be minimised to within an order of magnitude and investigate the potential of this strategy in view of quantum processing using exchange-based donor arrays.

## Results

### Valley interference and anisotropic envelope in exchange-coupled donors

In order to directly probe valley interference between donors, we have designed and fabricated samples to host isolated donor pairs embedded beneath a silicon surface (see Methods). Spatially resolved transport is performed at 4 K between a heavily doped reservoir and the tip of a scanning tunnelling microscope (STM). An STM image of a donor pair is shown in Fig. 1c. It is taken at a tip-sample bias of *V*_*b*_ = −0.95 V, close to the zero electric-field condition where the tip does not influence the two-electron neutral molecular state^7,38^. More specifically, the spatially resolved tunnelling current to the tip represents a quasi-particle wavefunction (QPWF), corresponding to the sum of the transitions from the two-electron ground state to energetically available one-electron states. Hence, the STM image contains two-electron wavefunction information including interference between the two donor wavefunctions^7,38,39^. Moreover, the exact site positions of the two donor ions (*P*_1_ and *P*_2_, respectively green and red dot) are determined out of different possible position configurations using a comprehensive image symmetry recognition protocol^40,41^, which notably include the tip orbital, as the oscillating pattern observed for each donor qualitatively varies depending on their position in the silicon lattice. In particular, donor *P*_1_ is located in the *z* = 5.5*a*_0_ atomic plane, in-between two dimer rows, and presents a minima at the surface projection of the ion location. The image corresponding to donor *P*_1_ presents two rows of 2 × 3 local maxima running along the $[1\bar{1}0]$ crystallographic axis, on each side of the ion location. On the contrary, donor *P*_2_ is found to be in the *z* = 6.75 *a*_0_ atomic plane, and sits directly underneath the middle of a dimer row of the 2 × 1 reconstructed surface. Donor *P*_2_ presents a maximum (instead of a minimum for *P*_1_) at this location, part of a row of three local maxima running along this dimer.

**Fig. 2:**
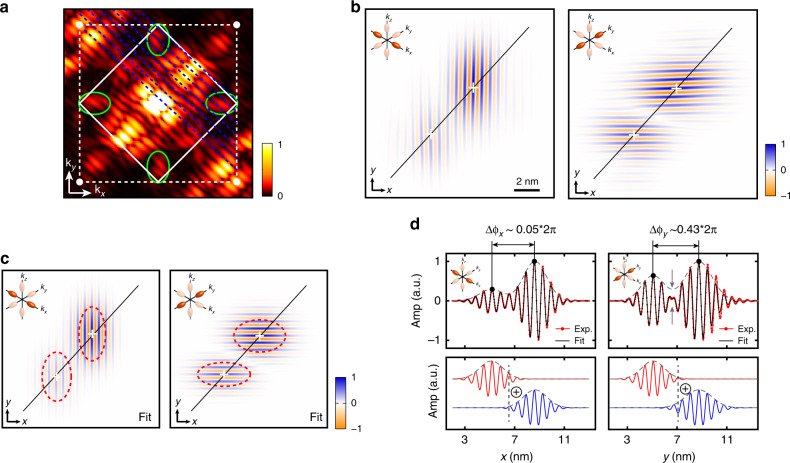
Visualising and quantifying valley interference between coupled donors. Pair #1. **a** 2D FFT of the STM image, centred on the first Brillouin zone (solid white lines). The white dots are located at  {±*k*_0_, ±*k*_0_}. The FFT shows a clear valley signal around *k*_*μ*_ ~ 0.81*k*_0_ evidenced by the green ellipsoids. The FFT also shows diagonal slices (blue dotted lines), cutting through the valley components of the FFT, which evidence the geometric interference between the two donors. **b** Real-space images of the valley interference, obtained after inverse Fourier transform of the FFT filtered around *k*_*x*_ ~ 0.81*k*_0_ for the *x*-valley interference (left) or *k*_*y*_ ~ 0.81*k*_0_ for the *y*-valley interference (right). The white crosses indicate the donor positions. The *x*-valleys look in-phase with the vertical stripes running continuously from one donor to the other. The *y*-valleys look out-of-phase with a clear discontinuity in the oscillatory valley pattern between the two donors. **c** 2D fits of the valley images, from which the position of the valley maxima and of the valley frequency, and hence the valley phase differences Δ*ϕ*_*x*_ and Δ*ϕ*_*y*_, can be obtained. The red dashed ellipsoids correspond to the donor envelope part of the fits, which highlights their anisotropy. **d** Line cuts taken through the ion–ion direction of both valley images (red lines) and their fit (black lines), for the *x*-valleys (left) and the *y*-valleys (right). The grey dashed lines represent the envelope part of the fits only. The *x*-valleys are in-phase, which results in the maxima of valley signal to always reach the envelop part. On the contrary, the *y*-valleys are out-of-phase and the grey arrows in-between the two donors highlight the clear reduction of the valley signal compared to the envelope part as a result of the destructive interference.

The reduced symmetries of the STM image compared to a spherical 1*s*-orbital for atoms in the vacuum, and the alignment of its local maxima with the dimer rows at the silicon surface clearly indicate the presence of both lattice and valley frequencies in the donor wavefunctions. These frequencies can be probed in more detail in Fourier space^42^. A contour mask was first applied around the two donors to avoid the presence of spurious frequencies in the fast Fourier transform (FFT) from the image boundaries. The first Brillouin zone of the resulting Fourier transform is shown in Fig. 2a. The different Fourier components which can be observed correspond to scattering processes between valley and lattice wavevectors^42^. In particular, the frequencies of the silicon 2 × 1 surface reconstruction can be identified at (*k*_*x*_, *k*_*y*_) = { ±1, ±1}*k*_0_ (with *k*_0_ = 2*π*/*a*_0_), corresponding to the ${a}_{0}\sqrt{2}$ periodicity between the dimer rows, i.e. along [110], as well (*k*_*x*_, *k*_*y*_) = ±(0.5, 0.5)*k*_0_ corresponding to the ${a}_{0}\sqrt{2}/2$ periodicity along [110] between each pair of silicon atoms forming a dimer. The components of interest for the valley interference analysis are found around  ±(0.81, 0)*k*_0_ and  ±(0, 0.81)*k*_0_. They relate to valley scattering processes, respectively between the  ±*x* and  ±*z*-valleys, and the  ±*y* and  ±*z*-valleys. It is important to note that these valley processes can be completely dissociated from any lattice frequency-associated process, since they rely on the valley momentum only. Moreover, the Fourier transform presents clear diagonal slices (blue dashed lines), which match the geometric destructive interference condition between the two donors. Their position with respect to the valley signal gives a hint to whether the valley states are in or out-of-phase, if their corresponding signal in the FFT falls in-between or on one these diagonal stripes, respectively (see Supplementary Note 1). In order to focus on these valley interference processes, a Gaussian filter was applied to the FFT around the valley signal (see green ellipsoids in Fig. 2a) and transformed back to real space. The left image in Fig. 2b shows the *x*-component result of this filtering: a set of vertical stripes, corresponding to the valley oscillations in the *x*-direction can be observed for each donor, with the valley phase pinned at each donor site. For this particular inter-donor distance, the *x*-valleys look in-phase as the vertical stripes run continuously from one donor to the other. The same filtering procedure was performed for the *y*-valley real-space image. In contrast to the *x*-valley, the *y*-valleys look out of phase with a clear break in the continuity of the horizontal stripes in-between the donors and a succession of phase slips. In order to be more quantitative, these valley images can be fitted to the sum of two 2D envelopes oscillating at the same frequency. The fitted 2D images are shown in Fig. 2c. They give the same visual impression of the fits as that of the experimental images. Furthermore, the valley phase differences can be extracted from the fits, yielding *k*_*x*_ = (0.8077 ± 0.0001)^*^k_0_, *k*_*y*_ = (0.8039 ± 0.0001)^*^k_0_, Δ*ϕ*_*x*_ = (0.050 ± 0.001)^*^2*π* and Δ*ϕ*_*y*_ = (0.434 ± 0.001)^*^2*π*, which confirms that the *x*-valleys are in-phase and that the *y*-valleys are out-of-phase. We show in Fig. 2d line cuts through the two ions of each valley image, experimental ones and their respective fit, to notably highlight the clear reduction of the *y*-valley signal in-between the two donors compared to the sum of the envelopes of the two donors due to the destructive interference.

**Fig. 3:**
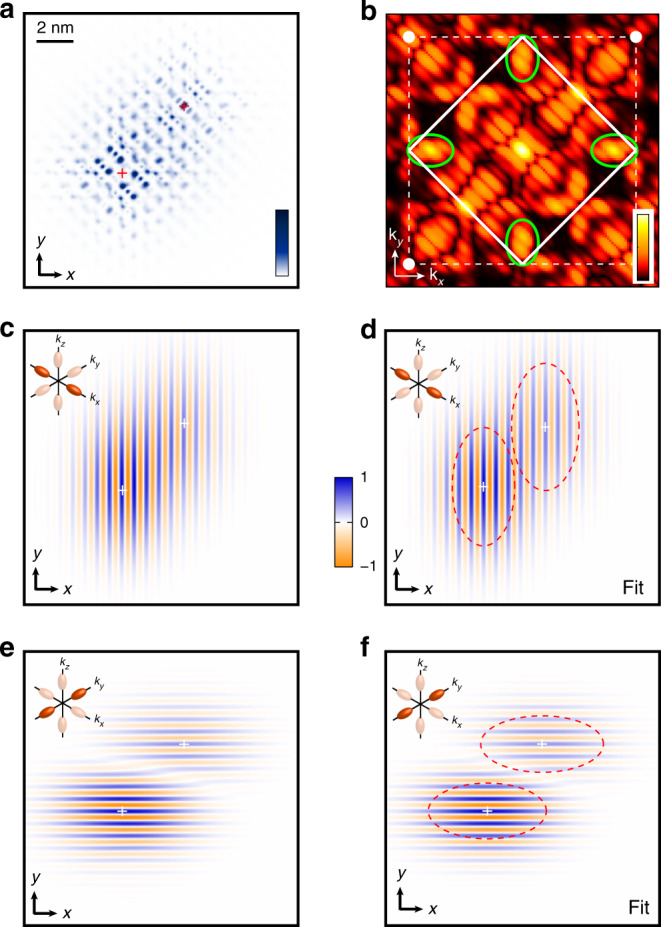
Theoretical two-electron STM image and valley interference. **a** Theoretical STM image computed using the pinpointed locations of the two donors and FCI calculations performed to determine the two-electron wavefunctions based on tight-binding one-electron states. **b** 2D Fourier transform of the STM image focussed on the first Brillouin zone. The valley components at 0.81*k*_0_ and the diagonal slices highlighting the interference are clearly visible. **c** Real-space image of the *x*-valley obtained by filtering the FFT around *k*_*x*_ ~ 0.81*k*_0_. **d** Corresponding fitted image, using the same fitting procedure as for the experimental data, and from which Δ*ϕ*_*x*_ and the ratio *b*/*a* for each donor (red dashed ellipsoids) can be extracted. As for the experiment, the *x*-valleys are found to be in-phase. **e**, **f** Same for the *y*-valleys, which like for the experiment are out-of-phase.

The procedure developed here establishes the existence of a geometric valley phase interference between donors, which depends on their relative position. This phase interference is by construction included in the Heitler-London regime of vanishing tunnel coupling, as the two electrons fully localise on the donors^7^. In this regime, the STM image becomes equivalent to the sum of the charge distributions of two independent single donors (see Fig. 2d and Supplementary Note 1). However, in our case the inter-donor distance is around 5 nm, at which some deviation from the Heitler-London regime is expected^33,34^, as highlighted by the finite overlap between the donor envelopes in Fig. 2b and d. To verify the deviation from the Heitler-London limit and whether the phase is robust is this regime where the electrons start to delocalise between the two donors, we have computed the STM image based on a calculated two-electron wavefunction which includes electron interactions. This theoretical image, shown in Fig. 3a, is computed using state-of-the-art atomistic modelling capabilities summarised here and detailed in the Methods section. First, starting from the donor pinpointed positions, the one-electron states are obtained by atomistic tight-binding (TB) modelling using a multi-million atom grid. The two-electron states are then obtained using a full configuration interaction (FCI)^36,43^ method based on 1*e*-molecular orbitals. Interface, reservoir and electric field effects are taken into account throughout this modelling framework to ensure an accurate description of the coupled donors spectrum. We found a two-electron ground state composed at 67% of the bonding *A*_1_–*A*_1_ ground state with an exchange energy of 1.5 meV (see Supplementary Note 1). As a reference, the Heitler-London limit results in an equal 50% contribution of both *A*_1_–*A*_1_ bonding and anti-bonding molecular orbitals since the tunnel coupling vanishes. The theoretical STM image is then obtained by computing the QPWF including STM transport and tip orbital effect^40^ and is shown in Fig. 3a. We have applied the same filtering procedure to obtain the FFT of this image, shown in Fig. 3b. It shows very similar features compared to the experimental FFT, with notably the presence of valley components at  ±0.81*k*_0_ and the diagonal slices which indicate the interference. The resulting *x*- and *y*-valley images are shown in Fig. 3c and d, respectively. The valley phases are also pinned to the donor ions and the valley phase differences can be obtained using an identical fitting procedure, giving $\Delta {\phi }_{x}^{{\mathrm{{FCI}}}}={(0.025\pm 0.001)}^{* }2\pi$ and $\Delta {\phi }_{y}^{{\mathrm{{FCI}}}}={(0.430\pm 0.001)}^{* }2\pi$, in good agreement with the experimental data as the fitted images in Fig. 3e and f show.

**Fig. 4:**
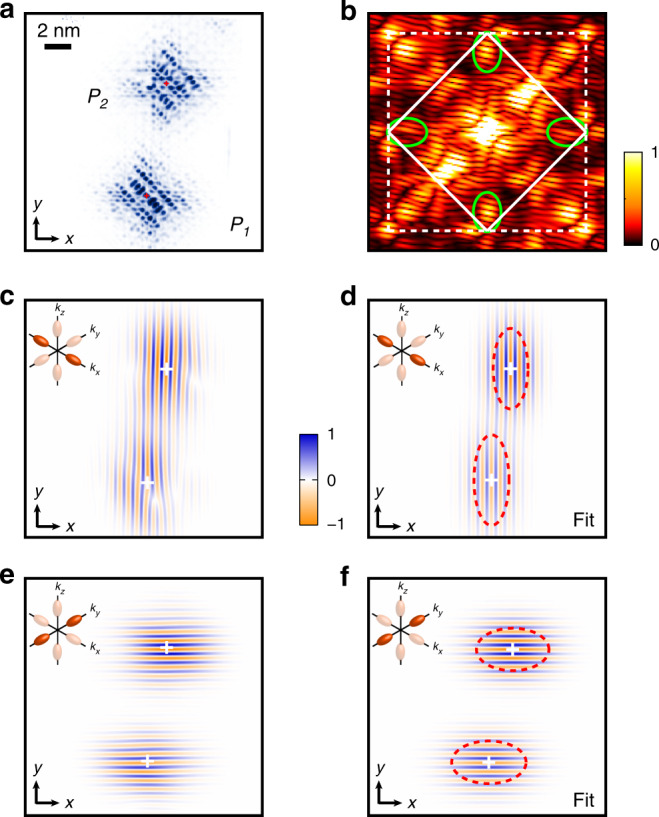
Valley interference for pair #2. **a** Experimental STM image of pair #2. The red crosses indicate the ion locations. **b** FFT of the STM image, centred on the first Brillouin zone. As for pair #1, the FFT shows a valley signal at 0.81*k*_0_ (green ellipsoids) and diagonal slices that indicate the interference, whose separation and slope is linked to the inter-donor distance and orientation. **c** Real-space image of the *x*-valley obtained by filtering the FFT around *k*_*x*_ ~ 0.81*k*_0_, same procedure as for pair #1. Same scale as the STM image shown in **a**. The observed distortions in the *x*-valley image, notably on the right-hand side of *P*_1_, are attributed to an instability of the tunnelling tip during the measurement. **d** Corresponding fitted image, using the same fitting procedure as for pair #1, and from which Δ*ϕ*_*x*_ and the ratio *b*/*a* for each donor (red dotted ellipsoids) can be extracted. **e**, **f** Same for the *y*-valleys.

**Fig. 5:**
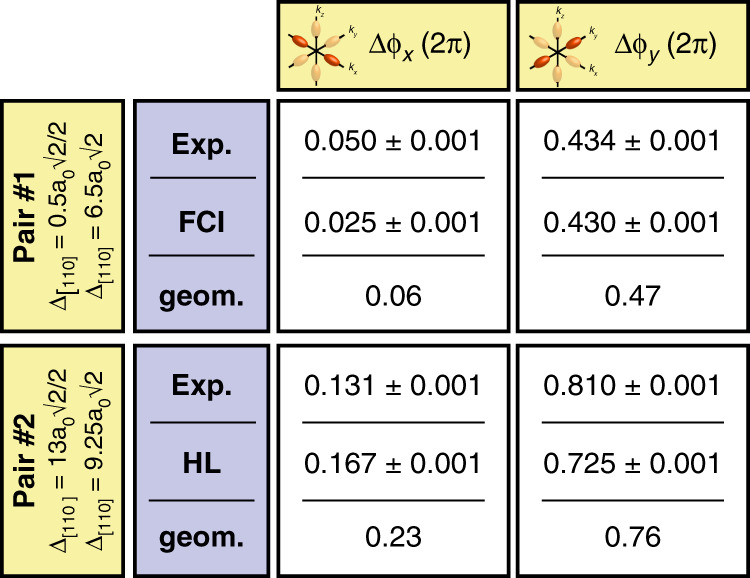
Table summarising the valley phase differences obtained for the two pairs. The *x*- and *y*-valley phase difference for each pair obtained from fitting the 2D valley images can be compared to the geometrical phase differences obtained from the pinpointed lattice separation between the two donors along [110] and $[1\bar{1}0]$ and assuming a valley momentum at 0.81*k*_0_.

We present in Fig. 4a the results obtained using the same protocol performed on another pair. The two donors are separated by $13{a}_{0}\sqrt{2}/2$ along [110] and $9.25{a}_{0}\sqrt{2}$ along $[1\bar{1}0]$, i.e. a larger inter-donor distance and a different orientation than the first pair. Its FFT (Fig. 4b) presents the same characteristics as for pair #1, with a clear valleys signal around 0.81*k*_0_ and the presence of diagonal stripes highlighting the geometric interference between the donors. The real-space images resulting from Fourier filtering around the valley signals and their respective fit are shown in Fig. 4c–f. We note the presence of phase distortions in the *x*-valley image which are different from a succession of phase slips expected for a destructive valley interference pattern. These isolated features are attributed to an instability of the tunnelling tip during the measurement (see Supplementary Note 1), and, importantly, they do not perturb the region of interest for the valley interference and the exchange interaction, i.e. the central region between the two donors. A theoretical STM image was also obtained and analysed, based on a Heitler-London calculation of the two-electron wavefunction as this pair shown an inter-donor distance >7 nm and therefore falls in this regime. We summarise the phase differences obtained for each pair in Fig. 5, both experimental and theoretical, to notably highlight the matching of the fitted valley phases to the geometrical phase difference expected from the exact lattice pinpointing of each donor atom. Full details on the filtering procedure, as well as on the robustness of the extracted valley phase differences against the dimension of the Fourier space filter, can be found in the Supplementary Note 1.

**Fig. 6:**
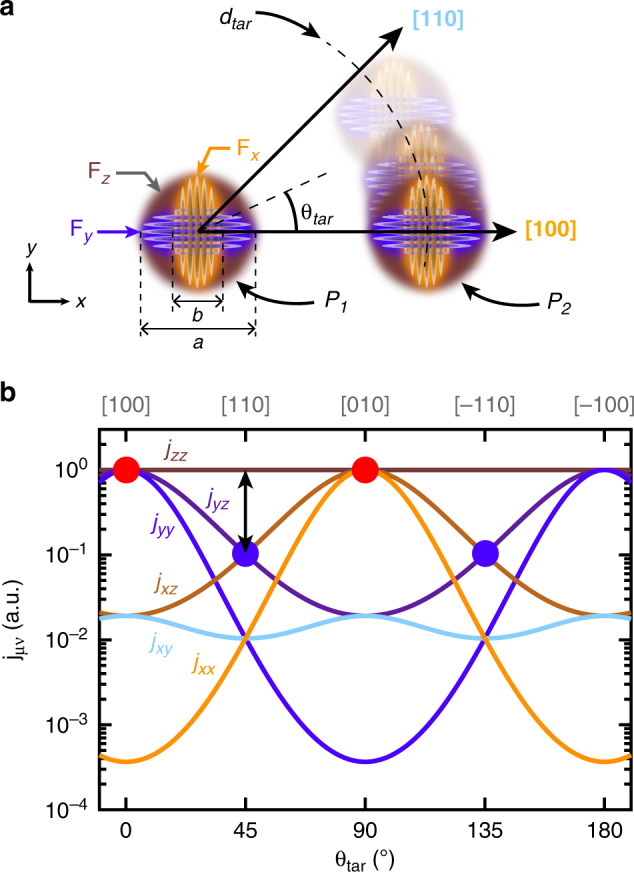
Donor envelope overlap and exchange interaction. **a** Two donors placed in the same *xy*-plane at a target distance *d*_tar_ and angle *θ*_tar_ defined from [100]. The exchange interaction can be considered as a sum of valley interfering envelope overlaps *j*_*μν*_ between the  ±*x*- (orange),  ±*y-* (purple) and  ±*z*-valleys (brown). The mass anisotropy results in each envelope orbital, *F*_*x*_, *F*_*y*_ or *F*_*z*_, to be constricted along its own direction. For instance, *F*_*x*_ has a small envelope radius *b* along *x*, and a large envelope radius *a* along *y* and *z*. **b** Normalised envelope weights *j*_*μν*_ plotted vs. *θ*_tar_ for *d*_tar_ =  12 nm, using *b*/*a* = 0.52 and *a* = 2.8 nm. Because of orbital anisotropy, the *j*_*yy*_, *j*_*yz*_ and *j*_*zz*_ terms (respectively *j*_*xx*_, *j*_*xz*_ and *j*_*zz*_ terms) are degenerate and dominate around [100] (respectively [010], red spots), while *j*_*zz*_ dominates around [110] and [−110] (blue spots).

The fitting procedure we have developed here also allows to investigate the envelope part of the donor valley states, whose characteristic extent are represented by the red dashed lines in Figs. 2–4, for each donor and each valley. Contrarily to a simple 1*s*-orbital with a spherical symmetry, the envelopes are here clearly ellipsoidal. This feature originates from the silicon mass anisotropy, which results in each valley orbital to present a small envelop radius *b* along their longitudinal direction and a large radius *a* along the two transverse directions^23,42^ (see Fig. 6a). The values *b*/*a* obtained across all experimental and theoretical image fits average to 0.52, which is in good agreement with single donors measurements^42^ (see Supplementary Note 1) and with other predictions^30,35,44^. Our experimental results demonstrate the existence of a valley interference effect between donor pairs which present a finite overlap between their wavefunctions. It is noteworthy that the tunnel current probes the extent of the wavefunction at the silicon/vacuum interface. In our case, this means that the tunnelling tip probes the overlap between the tails of sub-surface donor wavefunctions, which is precisely the essence of what the tunnel and exchange interactions rely upon.

### Exchange variations analysis for atomically precise donor qubit devices

Valley interference between neighbouring donors impact the exchange interaction in a non-trivial manner. To detail this effect, we have developed a model based on an effective mass Heitler-London formalism^30^. Importantly, this phenomenological effective mass (P-EM) model strongly relates to the valley phase differences Δ*ϕ*_*μ*_ and to the ratio *b*/*a* of the donor envelope which have been experimentally investigated above. The P-EM model decomposes the exchange interaction into a sum of oscillating terms, weighted according to six possible envelope terms *j*_*μν*_, as follows (see Supplementary Note 3):

1 $$J(\overrightarrow{R})=\sum _{\scriptstyle \mu ,\nu =\\ {\scriptstyle \pm \{x,y,z\}}}{j}_{\mu \nu }(\overrightarrow{R},a,b)\cos (\Delta {\phi }_{\mu }(\overrightarrow{R})\pm \Delta {\phi }_{\nu }(\overrightarrow{R})),$$

where *μ* (respectively *ν*) denotes the valley in which the first (respectively second) electron is exchanged between the two donors. Qualitatively, each weight *j*_*μν*_ is a four-term product, between two envelope orbitals *F*_*μ*_ separated by $\overrightarrow{R}$, and two envelope orbitals *F*_*ν*_, likewise also separated by $\overrightarrow{R}$. The 1*s*-like nature of the donor orbitals causes these envelope terms *j*_*μν*_ to vary exponentially with the inter-donor distance, with a characteristic length scale related to the major envelope radius *a*. As *k*_*μ*_ is close to *k*_0_, the valley phase differences evolve rapidly with site-to-site changes in the lattice position of the donors, which can strongly modulate the exchange. Both envelope and valley dependencies hence suggest that the exchange coupling will change significantly by the displacements of dopants, hence requiring atomic scale dopant placement accuracy^30,37^ for stability. To date the most precise dopant placement technique is obtained using STM lithography^5^, with the fabrication of in-plane atomic devices where donors can be placed within  ±1 lattice site precision. In the following, we investigate the exchange variations which can be expected using this unique device fabrication capability.

The relative weights of the six different envelope terms *j*_*μν*_ are plotted in Fig. 6b for a target distance *d*_tar_ of 12 nm as a function of the in-plane angle *θ*_tar_ defined with respect to [100]. To parametrise the P-EM model, the valley momentum and anisotropy ratio are set to the experimentally obtained average values, i.e. *k*_*μ*_ = 0.81*k*_0_ and *b*/*a* = 0.52. The large envelope radius *a* is set to 2.8 nm obtained from fitting Heitler-London calculations along [110] (see Supplementary Note 3). The weights (*j*_*xx*_, *j*_*xy*_, *j*_*xz*_, *j*_*yy*_, *j*_*yz*_ and *j*_*zz*_) evolve smoothly with *θ*_tar_ (and *d*_tar_), since they do not contain any valley interference, but their ratios are not constant and vary by several orders of magnitude. This is a direct consequence of the anisotropic and exponential nature of the donor envelope. Along [100], at *θ*_tar_ = 0^∘^, the *j*_*yz*_ terms are degenerate with *j*_*zz*_ and *j*_*yy*_, and largely dominate over any term where an electron is exchanged in a *x*-valley. This can be understood as mass anisotropy results in *F*_*x*_ to have a minor envelope radius *b* along *x*, hence the product between two *F*_*x*_ orbitals separated along [100] is smaller than its *F*_*y*_ and *F*_*z*_-orbital counterparts, as they both have a major envelope radius *a* along *x* instead. The degeneracy between *j*_*yz*_, *j*_*zz*_ and *j*_*yy*_ arises from symmetry as the products between *y* and *z* orbitals are equal along [100].

**Fig. 7:**
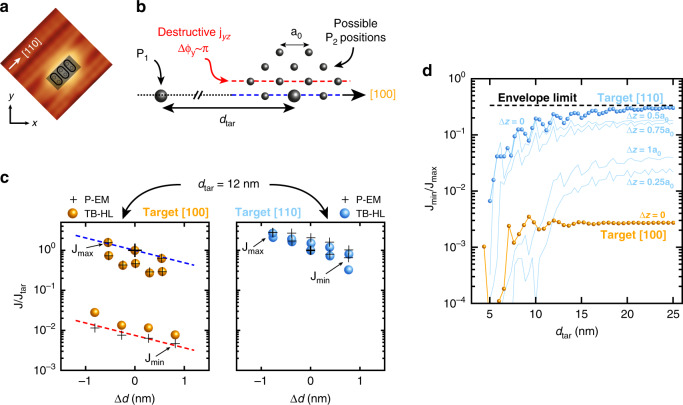
Limited exchange in-plane variations for STM placed donors. **a** Single donors can be incorporated in a patch of three desorbed hydrogen dimers on the silicon surface, giving six possible positions for each donor. **b** There are 12 non-equivalent position configurations for *P*_2_ for a target along [100], taking the convention to fix *P*_1_ at the origin. A target along [110] results in ten possible configurations. **c** Left: plot of the 12 possible exchange values along [100], normalised over the target one, plotted vs the distance difference Δ*d* = *d* − *d*_tar_ with *d*_tar_ = 12 nm, for both the P-EM (black crosses, the large one denotes the target) and TB-HL (orange balls) models. The configurations along the red dashed line (same as **b**) result in destructive *j*_*yz*_ terms and their exchange values are reduced by more than two orders of magnitude compared to the configurations perfectly aligned with [100] (blue dashed lines). Right: the exchange variations for a target along [110] are limited to less than a factor of 10 as *j*_*zz*_ dominates, making the exchange interaction insensitive to in-plane valley interference. **d** Ratios *J*_min_/*J*_max_ vs *d*_tar_ between 5 and 25 nm for both [100] (orange) and [110] (blue). The impact of in-plane valley interference vanishes for target distances beyond 12 nm along [110], to bound exchange variations to the envelope limit. Destructive *y*-valley positions, and the degeneracy between *j*_*yz*_, *j*_*yy*_ and *j*_*zz*_, are always present along [100] independently of the target distance, resulting in large exchange variations. The light blue lines show the exchange variations if the two donors are in different atomic *z*-planes, resulting in a finite *z*-valley phase difference.

The envelope weight anisotropy influences the way valley interference impact the exchange coupling. In order to get further insight on its role, we must consider the precise placement scheme provided by STM lithography. Each donor is stochastically incorporated within  ±1 lattice site precision in a patch of three desorbed hydrogen dimers (see Fig. 7a). This placement precision results along [100] in 12 non-equivalent configurations for the donors separation shown in Fig. 7b, taking the convention to fix P_1_ at the origin (see Supplementary Note 3). From pair to pair, different configurations will be stochastically obtained, resulting in the phase terms Δ*ϕ*_*x*_ and Δ*ϕ*_*y*_ to vary accordingly, and to potentially become destructive. Along [100], the degeneracy and dominance of the *j*_*yy*_, *j*_*zz*_ and *j*_*yz*_ terms implies that the sum expressed in Eq. (1) can be reduced to these terms, which only involve Δ*ϕ*_*y*_ and Δ*ϕ*_*z*_. Hence, Δ*ϕ*_*x*_ does not influence the exchange and can be ignored along [100]. Furthermore, Δ*ϕ*_*z*_ = 0 since the *z*-valleys are constructive anywhere in the *xy*-plane, and only Δ*ϕ*_*y*_ matters along [100]. The 12 values of exchange obtained from these configurations are plotted in Fig. 7c for a target distance of 12 nm. Assigning each of them to their respective position configuration allows to understand the observed spread. The values can be separated in four different groups according to their respective Δ*ϕ*_*y*_ since it is the only phase term of interest in this case. Among each group, the exchange values evolve monotonically since their valley phase terms are the same, leaving only an envelope dependence. The configurations perfectly aligned with [100] (blue dashed line in Fig. 7b, c) lead to constructive *y*-valleys, i.e. Δ*ϕ*_*y*_ = 0 and a maximised exchange coupling. The fast damping of exchange oscillations purely along [100] was already pointed out in previous work^30,35,44^, which can result in favouring [100] as the target axis if dopant placement accuracy is not appropriately considered. In fact, the configurations misaligned with [100] result in finite Δ*ϕ*_*y*_ and hence to a reduced exchange energy. In particular, in-plane positions off the [100] axis by *a*_0_/2, i.e. the closest positions to [100] (red dashed line in Fig. 7b, c), result in destructive *y*-valley interference and hence negative *j*_*yz*_ contributions to the exchange. These negative contributions almost perfectly cancel out the constructive *j*_*yy*_ and *j*_*zz*_ terms in the exchange equation, and the exchange energy is reduced by more than two orders of magnitude for these specific positions^30^. It is important to note that these destructive configurations are common to any previous theoretical work, although they had different conclusions because of different approaches in dopant placement accuracy (see Supplementary Note 3). Moreover, they are always present for any inter-donor distance since they result from the envelope weight degeneracy between *j*_*yy*_, *j*_*yz*_ and *j*_*zz*_ inherent to the [100] axis. These two arguments are crucial to definitely rule out [100] as a favourable placement axis.

Considering placement accuracy is essential when looking to fully understand the role of valley interference and envelope anisotropy on the exchange interaction. Some approaches have considered random dopant placement around a target^30,32,35,37^, but the actual in-plane placement accuracy at any in-plane angle *θ*_tar_ that STM lithography can ultimately offer has been overlooked. Away from [100] at finite *θ*_tar_, the envelope anisotropy breaks the degeneracy between the *j*_*yy*_, *j*_*zz*_ and *j*_*yz*_ terms (see Fig. 6b). The *j*_*zz*_ term becomes dominant over any term involving an electron exchanged in a *F*_*x*_ or *F*_*y*_ orbital, as the *F*_*z*_ orbitals are the only ones to face each other across their major envelope radius *a* independently of *θ*_tar_ (as their minor envelope radius is out of the plane). The prevalence of *j*_*zz*_ is maximised at *θ*_tar_ =  45°, i.e. for donors separated along [110]. There, a different symmetry condition is reached with a degeneracy occurring between the *F*_*x*_ and *F*_*y*_ orbital products since *x* = *y* along this direction. The following order is hence obtained, with the *j*_*zz*_ terms dominating over the degenerate *j*_*xz*_ and *j*_*yz*_ terms, themselves dominating over the *j*_*xx*_, *j*_*xy*_ and *j*_*yy*_ terms, which are also degenerate by symmetry. Placing donors along the [110] orientation results in finite Δ*ϕ*_*x*_ or Δ*ϕ*_*y*_, which can vary according to the configuration specifically obtained from pair to pair, and hence to destructive *j*_*xz*_ or *j*_*yz*_ terms. However, they both have a negligible impact on the value of exchange since *j*_*zz*_ dominates. As a result, the possible exchange values obtained for two donors aimed along the [110] axis are much more constrained and vary by less than an order of magnitude as shown in Fig. 7c. To summarise these results, we plot in Fig. 7d the ratio *J*_min_/*J*_max_ defined for each target distance and orientation. Along [100], the exchange energy presents large variations independent of target distance as the degeneracy between *j*_*yy*_, *j*_*yz*_ and *j*_*zz*_ remains. However, along [110] the in-plane valley interference Δ*ϕ*_*x*_ or Δ*ϕ*_*y*_ do not impact the exchange coupling for target distances beyond 12 nm as the variations reach an asymptotic limit set by envelope considerations only (see Supplementary Note 3). As for the [100] case, this result is in agreement with previous work^30,35,44^ (see Supplementary Note 3). It is revealed here through an atomic-scale understanding of the interplay between envelope anisotropy, degeneracies, valley interference and dopant placement accuracy. Furthermore, the dominance of the *j*_*zz*_ envelope terms along [110] results in Δ*ϕ*_*z*_ to be the only relevant valley phase difference, and thus to the exchange variations to be arranged as a function of the depth difference, i.e. atomic planes, between the donors. The resulting variations with respect to the maximum in-plane exchange value are shown in Fig. 7d for depth variations included within  ±1*a*_0_. Only the *a*_0_/4 and 1*a*_0_ planes show a significant reduction of the exchange interaction. Moreover, for target distances beyond 10 nm, none of these planes lead to exchange variations as large as for the in-plane exchange variations obtained around the [100] direction, which are the largest within  ±1*a*_0_ variation in *z*-placement for this direction (see Supplementary Note 3).

We also compared the variations obtained from our P-EM model to Heitler-London calculations based on the tight-binding wavefunctions (TB-HL), for which we already demonstrated in Fig. 2 an excellent agreement with the experiment at the wavefunction level. The TB-HL exchange calculations shown in Fig. 7c confirm two orders of magnitude of exchange variations for donors placed along the [100] direction because of the presence of destructive *y*-valleys configurations. They also confirm that exchange variations can be reduced to less than an order of magnitude along [110]. A more detailed level of comparison between TB and P-EM models is discussed in the Supplementary Note 3. Hence, TB formalism accurately models the donor wavefunction’s details and can be further used to predict the properties of advanced donor-based quantum devices which notably include electric fields. Finally, our understanding of the interplay between valley interference, envelope anisotropy and atomic placement on the exchange interaction which we developed from our P-EM model can be tied back to the original pair which has been experimentally measured. In order to obtain a normalisation constant for the exchange energies, we have fitted the exchange energy obtained from TB calculations in the Heitler-London limit along [110] for the bulk case (see Supplementary Note 3). We notably obtain excellent agreement on the value of the valley momentum *k*_*μ*_ and on the anisotropy *b*/*a* with the experimental ones. Equipped with this calibration, we can predict an effective mass exchange value for pair #1, which we found to be 1.50 meV, in excellent agreement with the FCI value calculated and mentioned above. Furthermore, we have also computed a nearby case of pair #1, where donor *P*_1_ was brought from the *z* = 5.5*a*_0_ to the *z* = 6.5*a*_0_ atomic plane. This shift results for these two donors to have the same *x* and *y* coordinates but to be separated by Δ*z* = 0.25*a*_0_, which is a very destructive plane difference for the *z*-valleys as seen in Fig. 7d for such pair in the neighbourhood of [110]. Indeed, the P-EM model yields an exchange of 0.133 meV and the FCI calculations 0.108 meV, again in excellent agreement with each other. We note that for such inter-donor distance, the *z*-arrangement of the exchange values along [110] is not fully formed yet, i.e. Δ*ϕ*_*x*_ and Δ*ϕ*_*y*_ are non-negligible for these two cases; nevertheless, this quantitative agreement clearly demonstrates the influence of the valley interference on the exchange interaction, as both values for pair #1 and its nearby cases are much lower than the maximum exchange values of the order of 10 meV that can be obtained in this neighbourhood.

## Discussion

The direct measurement and quantification of valley interference between donor states at the atomic-scale using STM, which was realised here, provides a detailed understanding of their impact on exchange variations^30,35,37^. Importantly, we found that the exchange interaction along the [110] direction is dominated by the *j*_*zz*_ term and is hence insensitive to in-plane valley interference Δ*ϕ*_*x*_ and Δ*ϕ*_*y*_. The agreement between atomistic calculations and the P-EM model, which relies upon parameters which we have here assessed experimentally, further establishes the importance of the envelope radius anisotropy and of valley interference to fully understand the behaviour of the exchange interaction at the atomic scale. Using the current state of the art STM lithography^5,18,45^ with  ±1 lattice site precision, the preferential crystallographic placement along [110] can be leveraged together with an in-plane placement resulting in Δ*ϕ*_*z*_ = 0. As a result, STM lithography enables to engineer donor devices where the exchange can be totally immune to valley interference, hence achieving a true semiconductor vacuum where complex degeneracies of the band structure can be ignored for coupled donors. The exchange variations are minimised to a factor of <10 between configurations where dopants are moved by  ±1 lattice site. This factor is only due to the change in the envelope overlap between the wavefunctions as it is the case for vacuum systems. Importantly, by symmetry, and as confirmed by our results in Fig. 6b, the exchange coupling along the [−110] direction (perpendicular to [110]) is also dominated by *j*_*zz*_ and therefore also protected from in-plane valley interference. As such it is possible to place donors using STM lithography along both the [110] and [−110] directions to create 1D and 2D arrays with reduced exchange coupling variations between nearest neighbours. In our work, the donors were found at different atomic planes, which we attribute to an annealing step at 600 °C performed during sample fabrication in order to flatten the surface for tunnelling spectroscopy purposes. Experimental progress has been made to minimise the segregation of highly doped phosphorus monolayers^46,47^, which should be further reduced for single donors as segregation and diffusion constants strongly depend on dopant concentration^48^. Single donor segregation mechanisms are predicted to activate from 250 °C^49^, which bulk donor qubit device fabrication can withstand^6^. These results motivate our scheme to keep the donors in the same plane around the [110] axis to maximise the exchange interaction uniformity.

Controllable exchange coupling between atom qubits is a key requirement for two-qubits gates, which must be performed with high fidelities and high speed in views of fault-tolerant quantum computing architectures^50,51^. Exchange variations from pair-to-pair can create rotation errors as the CNOT gate length is calibrated to a particular target value, which can impair the operation of a quantum processor. To avoid these errors, a composite CNOT gate can be performed with a so-called BB1 sequence instead of a single pulse^32,52^. This composite CNOT gate can be decomposed in a set of single and two-qubit rotations, with the advantage to maintain a high-fidelity above 99.9%, i.e. above quantum error correction thresholds, for any qubit pair despite a 10% error in the characterisation of the exchange coupling. For donors separated by 12 nm along the [110] direction, our TB-HL calculations give exchange values ranging from 11 to 93 MHz. These values can be used together with known single qubit gate times (340 ns^53^) to obtain CNOT gate times varying by <1% across an array (see Supplementary Note 4), with an average value of 1.2 μs, for fully characterised exchange values. This average CNOT gate time is dominated by the single qubit rotation time for this target distance, and the spread could be overcome by tuning the exchange values with each other electrically^36^. Such tuning would be impossible for donors placed along [100] because of too large exchange variations. The operation times expected for the out-of-plane configurations within one monolayer do not exceed 1.34 μs. For exchange values characterised to 90%, adding the correcting sequence to maintain the fidelity would result in an average CNOT operation time of 2.3 μs with a spread limited to 4% for the in-plane configurations, and a maximum time of 3.7 μs for out-of-plane configurations with only 6 out of 82 total configurations exceeding 3 μs. Whilst these operation times are slower compared to other spin-based CNOT gates^54–56^, they are well below donor coherence times which can approach seconds using dynamical decoupling sequences^57^.

As already mentioned, exchange and tunnel interactions are closely related since they both rely on wavefunction overlap considerations. From our exchange analysis, we obtain tunnel coupling values varying by less than a factor 5 for inter-donor distances along [110] down to 5 nm (see Supplementary Note 3). This distance is the lower bound at which the Heitler-London description is valid, as tunnel coupling values are there predicted to exceed 10 meV, i.e. becoming a substantial fraction of the 47 meV charging energy. Expected tunnel coupling values^35^ up to target distances of 12 nm along [110] remain above 200 μeV for in-plane configurations, and above 60 μeV for out-of-plane ones, all much larger than the achievable electronic temperature of about 50 mK (i.e. about 5 μeV). This opens the way to the study of Fermi-Hubbard problems robust to this level of disorder, as for the case of dimerised chains^58,59^, with the competition between topological gaps originating from the TB picture^60–62^ and on-site interactions. The prospect to extend studies to the regime of strong tunnelling interactions and to 2D systems makes donors in silicon a standout platform for quantum simulation of the Fermi-Hubbard model.

In summary, starting from direct real-space measurements of donor wavefunctions, we have been able to unambiguously quantify valley interference between donor atoms in silicon in real space and validate the predictions of existing theories. Driven by this experimental approach, we consider the dopant placement precision offered by STM lithography for a detailed understanding of the interplay between valley interference and envelope anisotropy on the exchange interaction. In full agreement with previous theoretical work, our results identify the [110] crystallographic direction as optimal for building 2D donor arrays where we predict less than an order of magnitude variation in exchange and tunnel couplings. We envision this fabrication strategy, in conjunction with quantum control schemes^32^ and exchange tuning mechanisms^36^, to be a key component in leveraging the exceptional coherence of donor qubits in silicon towards scalable quantum simulators and quantum processors.

## Methods

### Sample preparation

Samples were prepared in ultrahigh vacuum (UHV) with a pressure lower than 10^−10^ mbar, starting from a commercial n-type As-doped wafer with a resistivity of 0.001–0.003 Ω cm. Samples are first flash annealed three times around 1150 °C for a total of 30 s. After the final flash anneal, the temperature was rapidly quenched to 800 °C, followed by slow (1 °C/s) cooling to obtain a flat 2 × 1 surface reconstruction. Under these conditions, a layer of  ~15 nm from the Si surface is depleted from As dopants. P dopants are incorporated at this stage in Si by submonolayer phosphine (PH_3_) dosing with a sheet density of 5 × 10^11^ cm^−2^. This low-dose P *δ*-layer was overgrown epitaxially by  ~2.5 nm of Si. Growth parameters such as temperature and flux were chosen to achieve minimal segregation and diffusion whilst preserving a flat surface for STM imaging and spectroscopy purposes. Notably, the first nanometre is a lock-in layer grown at room temperature^46^. Subsequent growth alternates between 250 °C and 450 °C with a duration ratio of 3/1. A 600 °C flash follows for 10 s to flatten the surface. The surface is finally hydrogen passivated at 340 °C for 10 min under a flux of atomic H produced by a thermal cracker, in a chamber with a 10^−7^ mbar pressure of molecular hydrogen. STM measurements are taken in the single-electron transport regime described in refs. ^24,63^.

### Measurement techniques

The electrical measurements were carried out at 4.2 K in an STM (Omicron LT-STM). Both sample fabrication and measurements are done in UHV with a pressure lower than 10^−10^ mbar. The tunnel current *I* was measured as a function of the bias voltage *U* using ultralow noise electronics including a transimpedance amplifier. The differential conductance d*I*/d*U* shown in Supplementary Note 2 was obtained by numerical differentiation. Spatially resolved measurements of donor pairs quantum state were acquired using the multi-line scan technique, where the topography is recorded at *U* = −1.45 V during the first pass, and played during the second pass in open-loop mode with the current *I* recorded at the bias mentioned in the caption of the corresponding figures. The sample fabrication described above results in the donor pairs to be measured in the sequential transport regime, with a first tunnel barrier with tunnel rate Γ_in_ occurring from the highly doped substrate annealing, and the second tunnel barrier with tunnel rate Γ_out_ being a combination of the Si overgrowth after P deposition and the vacuum barrier, mainly dominated by the latter and tip-sample distance. Additional information regarding STM images and spectroscopy analysis can be found in the Supplementary Note 1.

### Atomistic simulations

Single-particle energies and wave functions for P donor in silicon are computed by solving a *sp*^3^*d*^5^s^*^ TB Hamiltonian^64^, where the P atom is represented by central-cell corrections including donor potential screened by non-static dielectric function^65^, and the P–Si nearest-neighbour bond-lengths are modified in accordance with the published DFT prediction^66^. The size of simulation domain (Si box) consists of roughly four million atoms with closed boundary conditions in all three spatial directions. The effect of surface strain due to 2 × 1 surface reconstruction is included in the TB Hamiltonian by properly displacing surface Si atoms and by modifying the inter-atomic interaction energies in the TB Hamiltonian^40^. The calculation of STM images of donor wave functions follows the published methodology^40^, where TB wave function is coupled with Bardeen’s tunnelling theory^67^ and derivative rule of Chen^68^. In our STM measurements, dominant contribution is from ${d}_{{z}^{2}}$ orbital in STM tip. For two-particle STM images, two-electron wave functions are computed from FCI approach^43^. The STM image represents a quasi-particle wave function resulting from 2e to 1e transition^39^. The resulting quasi-particle state is used to compute tunnelling matrix element described in the Supplementary Note 1. The exchange calculations shown in Fig. 7 are computed either from the corresponding atomistic TB single-particle wave functions^69^, or from a P-EM model detailed in the Supplementary Note 3, both based on Heitler-London formalism. XSEDE^70^, National Computational Infrastructure Australia and NanoHUB computing resources were used.

## Supplementary information

Supplementary Information

## Data Availability

Any data and code used for the purpose of this article are available upon reasonable request.
