# Supplementary material for: Valley interference and spin exchange at the atomic scale in silicon
Source: Nat Commun. 2020 Nov 30;11:6124. doi: 10.1038/s41467-020-19835-1 (PMC7705737; doi:10.1038/s41467-020-19835-1)
Supplement: Supplementary file 1 — Supplementary Information [file 41467_2020_19835_MOESM1_ESM.pdf]

Valley interference and spin exchange at the atomic scale in silicon.
-
Supplementary Information

B. Voisin[1†*], J. Bocquel[1†], A. Tankasala[2‡], M. Usman[3,4‡], J. Salfi[1], R. Rahman[2,5], M.Y. Simmons[1], L.C.L. Hollenberg[3], and S. Rogge[1*]
[1] *Centre for Quantum Computation and Communication Technology, School of Physics, The University of New South Wales, Sydney, 2052, NSW, Australia*
[2] *Electrical and Computer Engineering Department, Purdue University, West Lafayette, Indiana, USA*
[3] *Centre for Quantum Computation and Communication Technology, School of Physics, The University of Melbourne, Parkville, 3010 Victoria Australia*
[4] *School of Computing and Information Systems, Melbourne School of Engineering, The University of Melbourne, Parkville, 3010 Victoria Australia*
[5] *School of Physics, The University of New South Wales, Sydney, 2052, NSW, Australia*
[†‡] *These authors contributed equally*
**Corresponding authors: benoit.voisin@unsw.edu.au; s.rogge@unsw.edu.au*

Supplementary Note 1 - STM image analysis and comparison to TB-FCI theory

STM image correction and filtering

The STM image of the $2e$ to $1e$ transition is taken following the same procedure as in ref.[S1]: we perform a two-pass scan where, for each line of the scan, the topography is recorded during the first pass at large bias $U = -1.45\,\mathrm{V}$ (corresponding to imaging the valence band states of the silicon surface), and then played in the second pass with the STM tip feedback loop turned off and the bias plunged in the silicon gap at $U = -0.95\,\mathrm{V}$, above the $2e$-state transition of the two-donor system (see section S2).

The STM images analysis starts with a drift correction (Fig. S1a-b): a 2D-linear transformation is applied to the spatial coordinates in order to match the silicon lattice constant, using the topography image. The distance between the silicon dimers of the 2×1 reconstructed surface should equal $\mathrm{a}_0\sqrt{2}$, which is obtained via a 2D matrix transformation of the coordinates. We also apply an exponential correction to the tunnel current to compensate for the tip height variations present during the measurement, corresponding to the topography measured in the first pass.

A contour filter is applied to the corrected data, with data outside the yellow ellipse shown in Fig. S1c put to zero with a smooth transition around this contour to avoid artefact resonances in Fourier space. The 2D discrete Fourier transform, obtained numerically from the corrected and filtered data, is shown in Fig. S1d. The diagonal slices (blue dashed lines) arise from the Fourier transform of the sum of two similar objects shifted in space: let $f(\vec{r})$ be a given spatial function, like the STM image of a single donor, and $F(\vec{k})$ its Fourier transform. The Fourier transform of $f(\vec{r}) + f(\vec{r} - \vec{r_0})$ is then $F(\vec{k})(1 + e^{i\phi(\vec{k})})$ with $\phi(\vec{k}) = \vec{k}.\vec{r_0}$. The momentum positions $\vec{k_n}$ such as $\phi(\vec{k_n}) = \vec{k_n}.\vec{r_0} = n\pi$ result in the Fourier transform to go to zero, which can also be interpreted as a destructive interference condition. The position of the blue dashed lines which correspond to this condition with $\vec{r_0}$ being the relative donor-donor in-plane coordinates give a geometric hint to visualise the valley interference condition: the x-valleys, located at $\mathrm{k}_x \sim 0.81\mathrm{k}_0$, are in-between two slices and are therefore in-phase. On the contrary, the y-valleys around $\mathrm{k}_y \sim 0.81\mathrm{k}_0$ fall onto a blue diagonal slice and are then out-of-phase.

In order to quantitatively determine the valley phase difference, the data are multiplied by a Gaussian function in

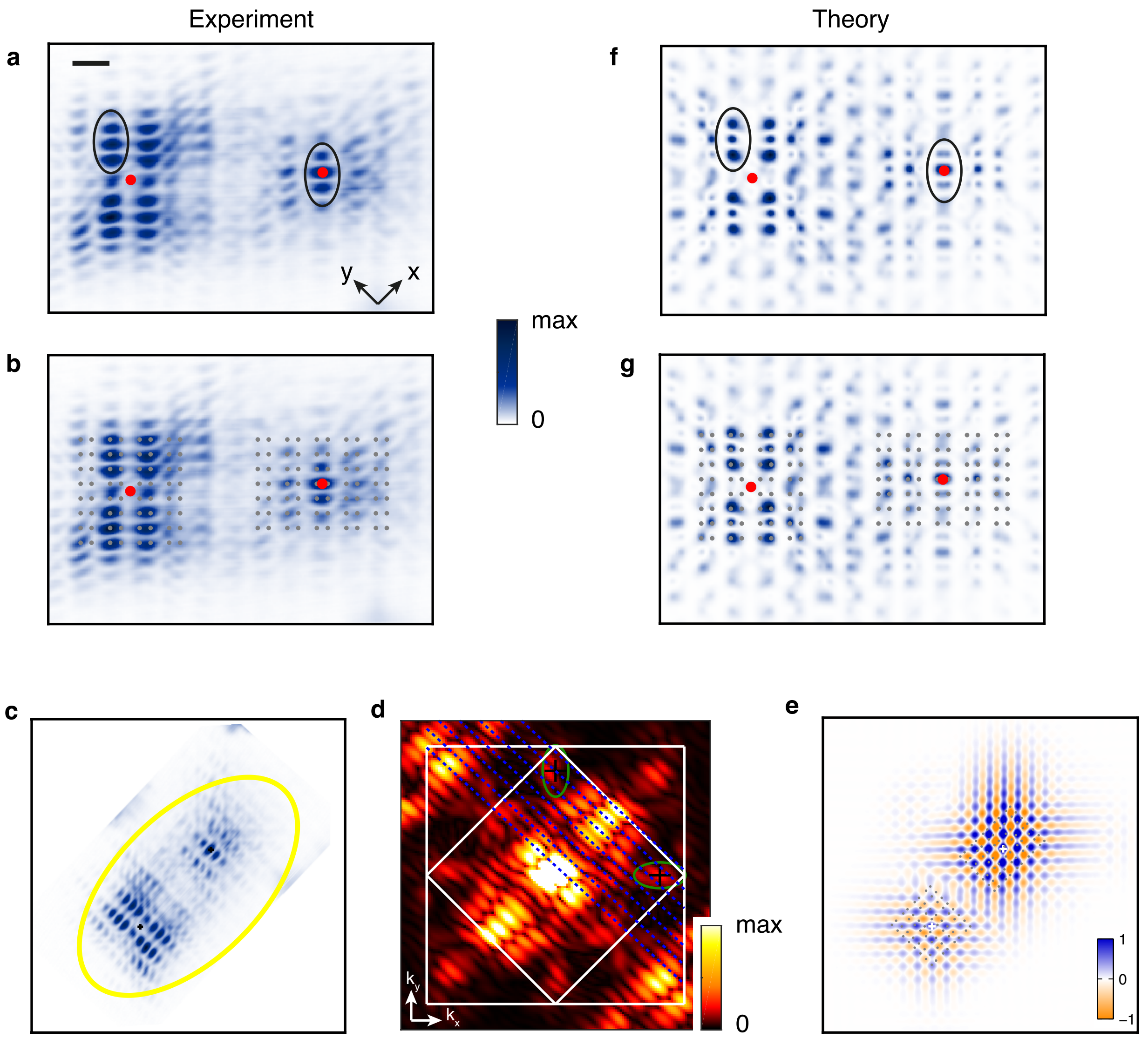

FIG. S1: **Drift correction and valley filtering in Fourier space. Pair #1. a,** Experimental STM image taken at U = −0.95V, after drift correction, same as in the main text (pair #1). Scale bar is 2 nm. The black ellipses indicate examples of features used to pinpoint the exact lattice site position of the phosphorus atoms, according to a procedure developed in ref[S2]. The red dots indicate the projection of the positions of the P atoms on the surface. **b,** same as **a**, with the grey dots indicating the positions of the silicon atoms of the 2×1 reconstructed surface. **c,** Experimental STM image. The yellow ellipse denotes the contour filter applied to the data before being Fourier transformed. Data is set to zero outside of the ellipse, preserved inside it. A low-passed filter is applied using a 3-point 2D Gaussian kernel to smoothen the transition between the two regions. **d,** FFT of the STM image (same as main text). The blue dashed lines represent destructive interference conditions, from which the interference condiition of the x and y-valley states can be deduced. **e,** Addition of the x and y-valley images shown in Fig.2d of the main manuscript. The grey circles represent the silicon atoms of the 2*1 reconstructed surface. The valley phases, i.e. maxima of the valley signal for each lobe, are pinned to the ion location of each donor, shown by the white crosses. The valley spatial period is clearly different from the lattice spatial periodicities, with also different orientation. **f-g** Same as **a-b** for the theoretical STM image computed from TB-FCI molecular state.

Fourier space (see equations S1 and S2). These Gaussian functions are centred around $k_x \sim 0.81k_0$ or $k_y \sim 0.81k_0$ to focus on the x or y-valleys, respectively. The filtered Fourier data are transformed back to real space to obtain the images shown in Fig. 2-3-5 of the main text. The variances f_r and f_t were carefully chosen in order to capture the relevant valley signal while avoiding other components of the Fourier transform as done in[S3], and their impact on the fits are discussed in detail below. The green ellipses in each Fourier image of the main text represent the 2σ-contour of the Gaussian filter masks which were used for both experimental and theoretical images, with f_r=0.1($2\pi/a_0$) and f_t/f_r=0.7. Back to real space, the amplitude $A_\mu(x,y)$ of each 2D valley image shown in the main text was fitted using the following equations S3 and S4 :

$$\text{mask}_x(k_x,k_y) = e^{-\frac{1}{2}[(\frac{k_x/(2\pi/a_0)-0.81}{f_r})^2+(\frac{k_y/(2\pi/a_0)-0.81}{f_t})^2]} \tag{S1}$$

$$\text{mask}_y(k_x,k_y) = e^{-\frac{1}{2}[(\frac{k_y/(2\pi/a_0)-0.81}{f_r})^2+(\frac{k_x/(2\pi/a_0)-0.81}{f_t})^2]} \tag{S2}$$

$$\begin{aligned}\text{Fit1}_x(x,y) = {} & A_1 e^{-(\frac{x-x_1}{\sqrt{2}b_1})^2-(\frac{y-y_1}{\sqrt{2}a_1})^2} * e^{-\frac{(x-x_1)^2+(y-y_1)^2}{2a_1^2}} * \cos\left(\frac{2\pi}{\lambda_x}(x-x_1)\right) + \\ & A_2 e^{-(\frac{x-x_2}{\sqrt{2}b_2})^2-(\frac{y-y_2}{\sqrt{2}a_2})^2} * e^{-\frac{(x-x_2)^2+(y-y_2)^2}{2a_2^2}} * \cos\left(\frac{2\pi}{\lambda_x}(x-x_2)\right)\end{aligned} \tag{S3}$$

$$\begin{aligned}\text{Fit1}_y(x,y) = {} & A_1 e^{-(\frac{x-x_1}{\sqrt{2}a_1})^2-(\frac{y-y_1}{\sqrt{2}b_1})^2} * e^{-\frac{(x-x_1)^2+(y-y_1)^2}{2a_1^2}} * \cos\left(\frac{2\pi}{\lambda_y}(y-y_1)\right) + \\ & A_2 e^{-(\frac{x-x_2}{\sqrt{2}a_2})^2-(\frac{y-y_2}{\sqrt{2}b_2})^2} * e^{-\frac{(x-x_2)^2+(y-y_2)^2}{2a_2^2}} * \cos\left(\frac{2\pi}{\lambda_y}(y-y_2)\right)\end{aligned} \tag{S4}$$

which gives access to the (x,y)-coordinates of the absolute maxima and to the anisotropy (b/a) for each donor and each valley, as well as a common valley wavelength λ_μ. The phase differences are obtained using $\Delta\phi_x = |x_2 - x_1|/\lambda_x$ and $\Delta\phi_y = |y_2 - y_1|/\lambda_y$, modulo 2π, and their corresponding confidence intervals are derived from the fit parameters deviations. Fig. S1e shows that the maxima of the sum of the x and y-valley image fall on the ion positions, which means that the valley phase is pinned to the ion for each donor.

Comparison to FCI theory

The theoretical STM image calculations protocol is described in the Methods section of the paper. The tunnelling matrix element between donor and tip states is defined as:

$$M = \frac{2}{3}\frac{\partial^2\Psi_D(r)}{\partial z^2} - \frac{1}{3}\frac{\partial^2\Psi_D(r)}{\partial y^2} - \frac{1}{3}\frac{\partial^2\Psi_D(r)}{\partial x^2} \tag{S5}$$

For two-particle STM images, two-electron wave functions are computed from a full configuration interaction approach[S4]. The STM image represents a quasi-particle wave function resulting from $2e$ to $1e$ transition (see below). The resulting quasi-particle state is used to compute tunnelling matrix element described above. The exchange interaction obtained from FCI (see below) is the energy difference between the ground and the first triplet state. The FCI $2e$ ground state is defined as a sum of Slater determinants:

$$|\Psi_s\rangle = \sum_{\alpha\beta} d_{\alpha\beta}\,|\alpha\beta\rangle \quad \textit{with} \quad |\alpha\beta\rangle = \frac{1}{\sqrt{2}}(|\alpha\rangle_1 |\beta\rangle_2 - |\beta\rangle_1 |\alpha\rangle_2) = c_\alpha^\dagger c_\beta^\dagger |0\rangle \tag{S6}$$

and $|\alpha\rangle$ are $1e$ molecular orbital (including spin). The tunnelling current, governed by $\Gamma_{\text{out}} \ll \Gamma_{\text{in}}$, can be expressed as the sum of the $2e$ to $1e$ transitions following[S5].

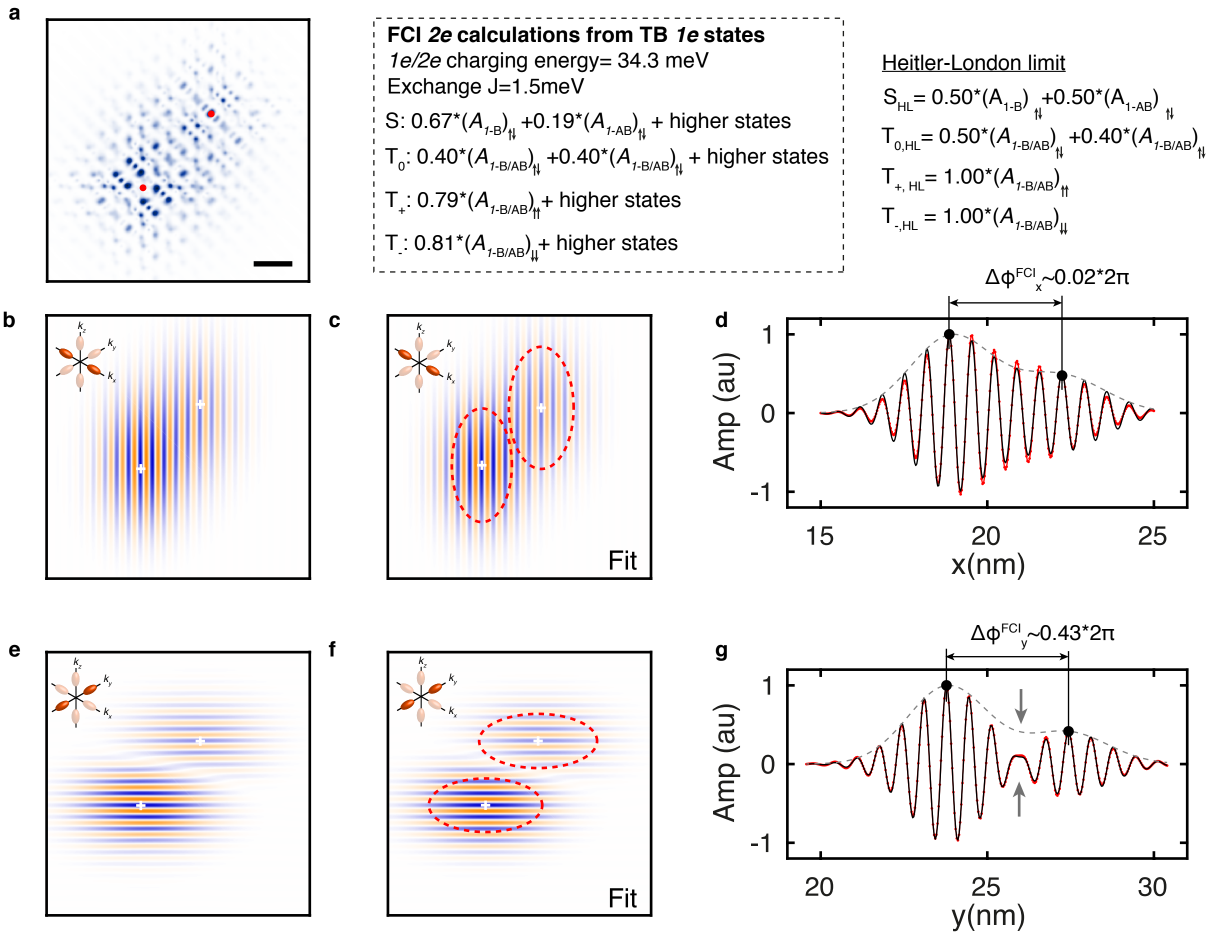

FIG. S2: **Details on FCI calculations and theoretical valley phase difference for pair #1.** **a,** Theoretical STM image (scale bar is 2 nm) based on 2e-FCI calculations, same as main text. Details are given about the charging energy, exchange energy and about the molecular orbital composition of the 2e singlet and triplet states. The comparison to a pure Heitler-London state is also given on the right-hand side. **b,** Theoretical STM image filtered around the x-valleys as shown in the main text. **c,** Fitted valley image using eq. S3, same as main text. **d,** Line cut taken across the two ions for both images. In-between the two donors, the amplitude of the valley signal remains at the maximum of the sum of the envelope of both donors (grey dashed line) as the x-valley interference are constructive. **e-g** Same as **b-d** for the y-valleys. The amplitude of the valley signal in-between the two donors is reduced compared to the sum of the two envelope as the y-valleys interfere destructively, as highlighted by the grey arrows.

$$\Gamma_{out}(\vec{r}) = \sum_i |M_{iS}(\vec{r})|^2 = \sum_i |\langle i| \Psi(\vec{r}) |\Psi_s\rangle|^2 \tag{S7}$$

with $|\mathrm{i}\rangle = \mathrm{c}_\mathrm{i}^\dagger |0\rangle$ and $|\Psi(\tilde{\mathrm{r}})\rangle = \sum_\mathrm{i} \mathrm{c}_\mathrm{i}\phi_\mathrm{i}(\tilde{\mathrm{r}})$ the tunneling field operator. We obtain:

$$\begin{aligned} M_{iS}(\vec{r}) &= \sum_{\alpha\beta} d_{\alpha\beta} \langle 0| c_i \sum_j c_j \phi_j c_\alpha^\dagger c_\beta^\dagger |0\rangle \\ M_{iS}(\vec{r}) &= \sum_{\alpha\beta} d_{\alpha\beta}(\phi_\alpha\delta_{i\beta} - \phi_\beta\delta_{i\alpha}) \end{aligned} \tag{S8}$$

The theoretical image is shown in Fig S1e-f. We can observe that the valley pattern symmetries for each donor are well preserved. The 2D Fourier transform of this image, shown in Fig3a of the main text also shows the valley signal at $k_{x,y} \sim 0.81 * k_0$ and the same stripes as for the experimental data.

Further details on the FCI calculations for this specific pair are given in Fig S2a. A charging energy of 34.3 meV and an exchange energy (taken as the energy difference between the first singlet (ground) state and the first triplet state) of 1.5 meV can be extracted from FCI calculations. Moreover, the FCI calculations gives the contribution of each molecular orbital in the $2e$ states. The FCI calculation singlet state yields a 67% contribution from the A_1 bonding state (called A_{1-B}), 19% contribution from the A_1 anti-bonding state (called A_{1-AB}), the rest coming from higher valley and orbital states, notably the T_2 bonding state which comes down in energy because of the tunnel coupling between the two donors[S6,S7]. We can discuss the deviation to a pure Heitler-London state $2e$ molecular state which would be made of the single donor A_1 ground states. The net dominant contribution of the A_1 bonding state for pair #1 comes for the finite tunnel coupling value: a pure HL state would result in a 50% contribution of both A_1 bonding and anti-bonding states, as these two orbitals are degenerate for vanishing tunnel coupling. A similar study can be done for the triplet case, which shows a 80% contribution of a combination of bonding and anti-bonding molecular orbitals (called $A_{1-B/AB}$) to form the triplet states. Finally, we show in Fig S2d and g a line cut taken across the two ions for both the x and the y-valley theoretical image in Fig S2b and e, respectively, as well as of their respective 2D fit, Fig S2c and f. Similarly to the experimental data, the y-valley signal in-between the two donors is weaker than the sum of the envelopes (grey dashed line in Fig S2g) because of the destructive y-valley interference.

STM image and Heitler-London regime

Let's consider only the two A_1 ground states for each donor. The 2e ground state can be expressed as a combination of even and odd combination of A_1:

$$\psi_S^{A1}(\vec{r}) = \gamma_{ee}\phi_{ee}(\vec{r}) + \gamma_{oo}\phi_{oo}(\vec{r})$$
$$with \quad \phi_{ee}(\vec{r}) = \frac{\psi_1^{A1}(\vec{r}) + \psi_2^{A1}(\vec{r})}{c_{ee}\sqrt{2}} \quad and \quad \phi_{oo}(\vec{r}) = \frac{\psi_1^{A1}(\vec{r}) - \psi_2^{A1}(\vec{r})}{c_{oo}\sqrt{2}} \tag{S9}$$

The associated expression for the tunneling current follows:

$$\Gamma_{out}(\vec{r}) = |\gamma_{ee}|^2|\phi_{ee}(\vec{r})|^2 + |\gamma_{oo}|^2|\phi_{oo}(\vec{r})|^2 \tag{S10}$$

In the Fermi-Hubbard framework the Heitler-London limit U/t $\rightarrow \infty$ leads to $\gamma_{\rm ee} = \gamma_{\rm oo} = 1/\sqrt{2}$[S8]. This results in the following STM current:

$$\Gamma_{out}^{HL}(\vec{r}) \propto |\phi_{ee}(\vec{r})|^2 + |\phi_{oo}(\vec{r})|^2 \propto |\psi_1^{A1}(\vec{r}) + \psi_2^{A1}(\vec{r})|^2 + |\psi_1^{A1}(\vec{r}) - \psi_2^{A1}(\vec{r})|^2$$
$$\Gamma_{out}^{HL}(\vec{r}) \propto |\psi_1^{A1}(\vec{r})|^2 + |\psi_2^{A1}(\vec{r})|^2 \tag{S11}$$

which is a $|{\rm D}_0(\vec{r}-\vec{r_1})|^2+|{\rm D}_0(\vec{r}-\vec{r_2})|^2$ STM image, i.e. the sum of the images of two single donors. This limit makes an evident link between the $2e$ STM image and the $1e$ probability density for each donor, which hence contain both the geometric valley interference terms between the two donors. As it can also be deduced between the Heitler-London relationship $J = 4t^2/U$ between the tunnel coupling t (a $1e$ quantity) and the exchange coupling J (a $2e$ quantity), valley interference impact both $1e$ and $2e$ processes.

2nd pair valley analysis

Here we present complimentary data for pair #2, where the donors are found to be distant by $13{\rm a}_0\sqrt{2}/2$ along [110] and $9.25{\rm a}_0\sqrt{2}$ along $[1\bar{1}0]$. Donor P_1 is found at z=6.5a_0 and P_2 at z=6.25a_0. We show the surface lattice superimposed on the STM image in Fig. S3b. The yellow contour shown in Fig. S3a shows the filter applied on the data before the Fourier transform. For this pair, the theoretical image was computed using the Heitler-London limit for the 2e ground state as reference, which is relevant for the inter-donor distance d>7 nm measured in this case. The resulting theoretical image STM image is shown in Fig. S3c and d, without and with the lattice superimposed, respectively. We note the excellent agreement between the features of the images along and across the dimers between the experimental and theoretical images, which are used to pinpoint the donors location[S2], as it was done for pair #1. The Fourier transforms of the images are shown in Fig. S3g and h, for the experimental and the theoretical image, respectively. Again, we note a good agreement on the features present in the first Brillouin zone, with only

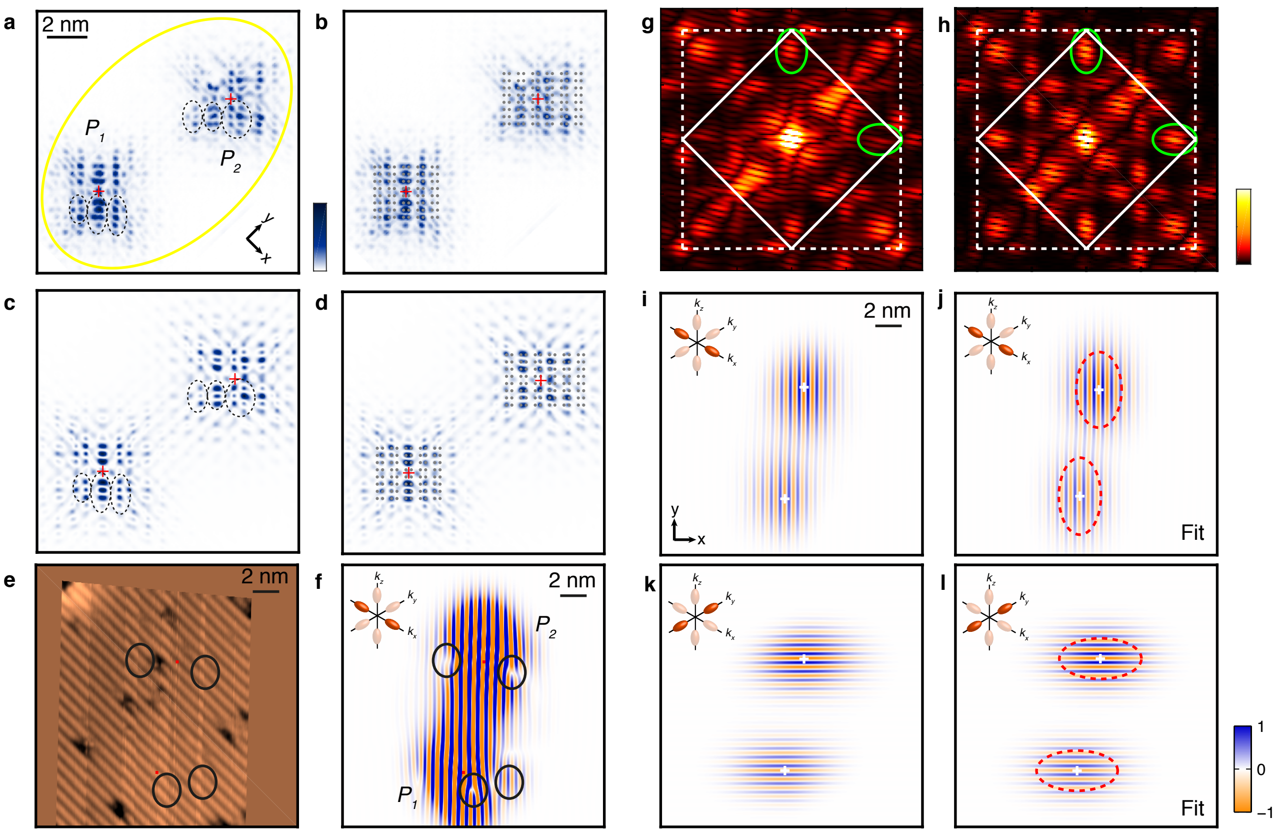

FIG. S3: **Valley analysis for pair #2. a,** Experimental STM image. The yellow ellipse represents the contour filter used before the Fourier transform. **b,** Experimental image with the surface lattice superimposed. Donor P_1 sits in the middle of a dimer row, in-between two pairs of atoms forming a dimer. Donor P_2 sits underneath a row of silicon atoms, in-between two dimer pairs. **c,** Corresponding theoretical STM image calculated from a Heitler-London 2e states, i.e. a $|\mathrm{D}_0|^2 + |\mathrm{D}_0|^2$ image. The black ellipses in **a** and **c** point to instances of matching features between the two images which were used to pinpoint the donors location. **d,** Same as **c** with the surface silicon lattice superimposed. **e,** Experimental topography taken at $U = -1.6\,\mathrm{V}$. There are evident tip jumps circled in black in the measurement along the x-axis (slow scan). **f,** 2D x-valley image, same as main text only with a saturated color scale, in order to focus on the forks and distortions of the valley oscillations. The black circles from **e** placed at the same locations evidence for the tip jumps to be origin of the distortions in the valley oscillations. **g,** Experimental Fourier transforms based on **a**. **h,** Theoretical Fourier transform based on **c**. **i,** Resulting x-valley image from Fourier filtering around the conduction band minima with f_r=0.1$(2\pi/a_0)$ and f_t=0.7f_r. **j,** Fit of the x-valley image using eq. S3. **k,l** Same as **i,j** for the y-valleys. The fit parameters shown in the tables of the main text for pair #2 originate from these images.

a weaker signal for the x-valleys in the case of the experiment, which we believe is due to the jumps in the tunnel current while the STM image was being measured with a slow scan along this x-axis. These jumps are best seen in the topography pass, as shown in Fig. S3e. The black circles on this topographic image run over some of these tip jump, and they match the forks, or distortions, seen in the x-valley image of the experimental data shown in the main text, and reproduced and amplified with a saturated colorscale here in Fig. S3f. To complement the data shown in the main text regarding pair #2, we show here in Fig. S3i-l the x and y-valley images as well as their fit for the theoretical STM image, which correspond to the phase differences shown in Fig.5 of the main text and to the envelope radius anisotropy shown in the table below.

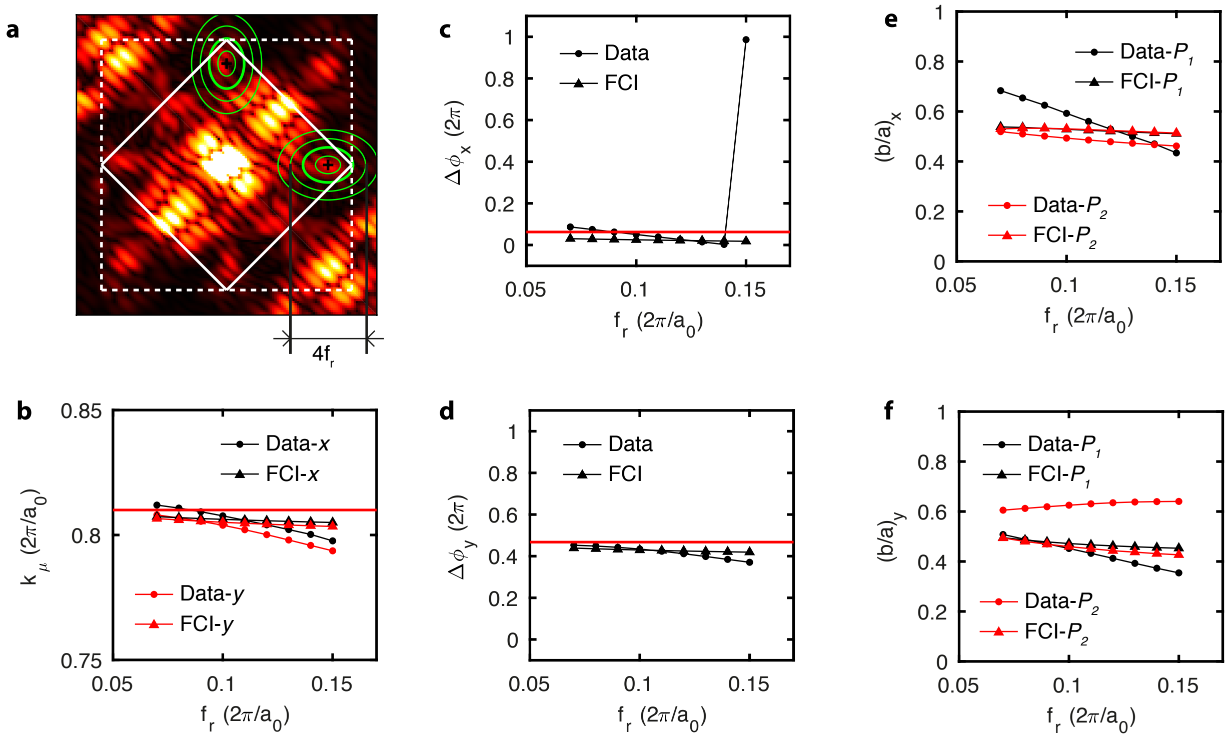

FIG. S4: **Analysis of the fit parameters vs filter dimension for pair #1. a,** FFT of the experimental STM image. The fitting procedure was performed for different Fourier filter dimensions f_r values in S1 and S2, with f_t=0.7f_r. f_r=$\{0.05, 0.10, 0.15, 0.20\}(2\pi/a_0)$ from the inner to the outer green ellipsoids around the valley signal at $k_{x,y} = 0.81k_0$, respectively. **b,** Valley momentum obtained vs f_r for both the experiment (dots) and FCI calculations (triangles), and both x (black) and y-valleys. The red line corresponds to k_μ=0.81k_0. The values change by less than 2% over the range of f_r which was studied. **c,** $\Delta\phi_x$ vs f_r for the experiment and FCI. The red line at $0.06(2\pi)$ corresponds to the predicted geometric interference condition from the donors lattice position and k_μ=0.81k_0. Both experimental and theroretical values remain very close to the geometrical interference condition. The experimental point for f_r=0.15$(2\pi/a_0)$ is simply shifted by a modulo $[2\pi]$. **d,** Same for $\Delta\phi_y$. **d,** Anisotropy b/a obtained for the x-valleys, for both experiment (dots) and FCI (triangles), for both P_1, i.e. b_1/a_1 in eq. S3 (black) and P_2, i.e. b_2/a_2 in eq. S4 (red). The experimental anisotropy for P_1 shows a larger dependence with f_r, while the others are close to the effective mass value of 0.52. **e,** Same for for the y-valleys.

Robustness of the valley phase difference to the dimension of the Fourier filter.

Choosing an appropriate filter in Fourier space is crucial for our analysis, and a trade-off must be considered. On one hand, the filter should not be too small as it would restrict the range of frequencies which could be obtained and enforce a single value. Moreover, using a filter whose k-space extent is smaller than the feature of interest would result in an artificially enlarged feature in real-space, and the result envelop radii and anisotropy could not be trusted. On the other hand, the filter cannot be too large as it would eventually include other Fourier components than that of interest, which would alter the fitting procedure in real space and the relevance of the results. We show in Fig. S4a the FFT for pair #1 and a range of different ellipsoids around the x and y-valley components, with respectively $f_r = \{0.05, 0.1, 0.15, 0.2\}(2\pi/a_0)$ from the inner to the outer ellipsoid, and f_t/f_r=0.7. Clearly the ellipsoid with f_r=0.05$(2\pi/a_0)$ does not englobe the whole valley signal, while f_r=0.15$(2\pi/a_0)$ leaks out to other components of the Fourier image. Therefore we estimate that a range from f_r=0.07$(2\pi/a_0)$ to f_r=0.15$(2\pi/a_0)$ is appropriate to analyse this valley component. The figures presented in the main text correspond to images and fitting parameters all obtained with f_r=0.10$(2\pi/a_0)$ and f_t=0.7f_r.

We show in Fig. S4b the evolution of k_μ with f_r, for both experimental and FCI data, and both x and y-valleys. A small dependence can be observed for the experimental values, however it is remarkable that all the values are found to vary by less than 2% over the considered range of f_r, which is extremely restrained considering the range of frequencies which has become available for f_r=0.15$(2\pi/a_0)$. The resulting dependence for $\Delta\phi_x$ and $\Delta\phi_y$ are shown

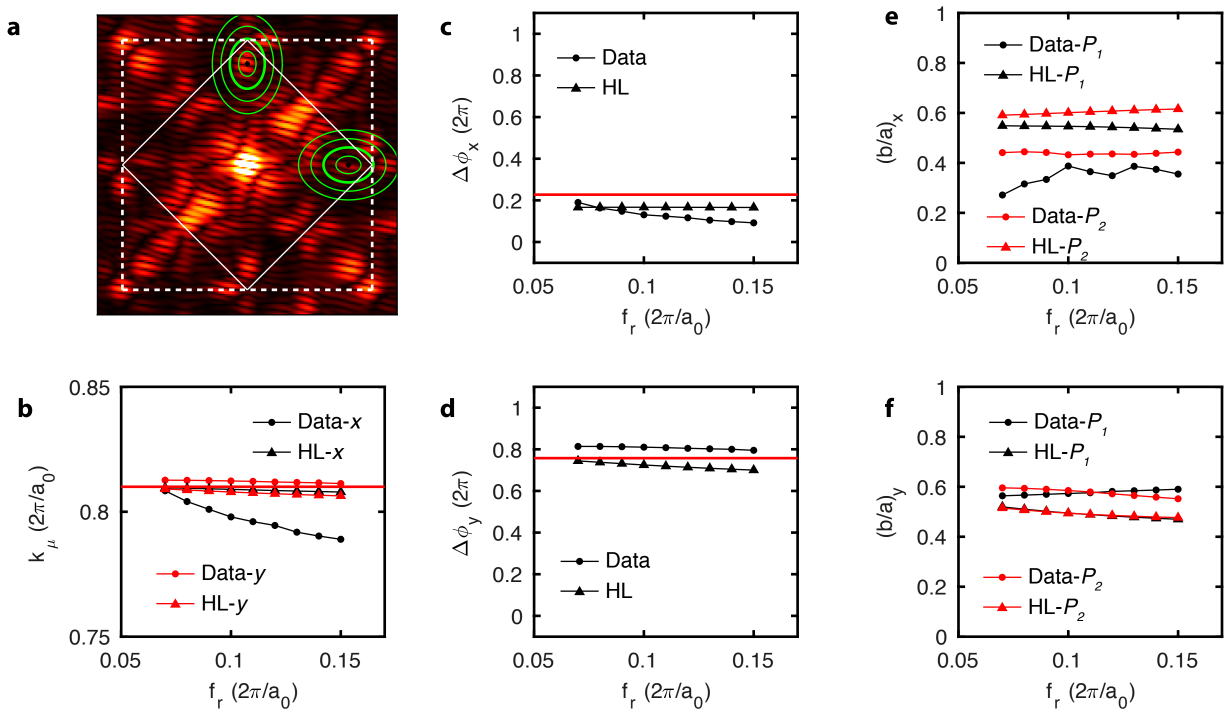

FIG. S5: **Analysis of the fit parameters vs filter dimension for pair #2. a,** FFT of the experimental STM image. The fitting procedure was performed for different Fourier filter dimensions f_r values in S1 and S2, with f_t=0.7f_r. f_r=$\{0.05, 0.10, 0.15, 0.20\}(2\pi/a_0)$ from the inner to the outer green ellipsoids around the valley signal at $k_{x,y} = 0.81k_0$, respectively. **b,** Valley momentum obtained vs f_r for both the experiment (dots) and FCI calculations (triangles), and both x (black) and y-valleys. The red line corresponds to k_μ=0.81k_0. The 2% deviation of experimental value for k_x from 0.81k_0 is attributed to the tip jumps associated with the measurement. **c,** $\Delta\phi_x$ vs f_r for the experiment and FCI. The red line at $0.23(2\pi)$ corresponds to the predicted geometric interference condition from the donors lattice position and k_μ=0.81k_0. **d,** Same for $\Delta\phi_y$. **d,** Anisotropy b/a obtained for the x-valleys, for both experiment (dots) and FCI (triangles), for both P_1, i.e. b_1/a_1 in eq. S3 (black) and P_2, i.e. b_2/a_2 in eq. S4 (red). The values, ranging from 0.4 to 0.6 apart from the experimental value for P_1 due to the tip jumps, are close to the effective mass value of 0.52. **e,** Same for for the y-valleys. Again the values are on average close to 0.52.

in Fig. S4c-d, respectively. The phase differences remain very close to the predicted geometric interference, with also very limited spread which can be related to the dependence in k_μ. The ratio in the envelope radii, shown in Fig. S4e-f, fir the x and y-valleys respectively, consistently remains below 0.65, which evidences a clear anisotropy. We note that the x-valleys of P_1 shows a stronger dependence of the ratio with the filter dimension than the other values. We have performed the same procedure for pair #2, with the results shown in Fig. S5. We obtain very similar robustness of the phase differences anisotropy as for pair #1. We note that an enhanced dependence of k_x with f_r, although still limited to 2%, which we attribute to the instability of the tunneling tip as explained above.

		P_1 b/a	P_2 b/a
Pair #1	Exp.	0.593±0.004	0.493±0.001
	FCI	0.529±0.001	0.530±0.001
	Exp.	0.452±0.003	0.625±0.002
	FCI	0.472±0.001	0.459±0.001
Pair #2	Exp.	0.387±0.003	0.432±0.002
	HL	0.546±0.001	0.601±0.001
	Exp.	0.573±0.001	0.585±0.001
	HL	0.495±0.001	0.494±0.001

FIG. S6: **Envelope anisotropy fitting results.** Table summarising the anisotropy ratios b/a for each donor, obtained from the fit of the valley images for the two pairs, for both experimental and theoretical images. The values average to 0.52 in agreement with single donors measurements and calculations.

Envelope anisotropy

We show in Fig. S6 the anisotropy ratios b/a, as well as standard deviations, obtained from fitting the 2D filtered images according to equations S3 and S4. Each donor of each pair gives a value for the x and the y-valleys, for both experimental and theoretical images, which results in a total of 16 values. The values show a spread from 0.387 to 0.625, which clearly establishes the existence of an envelope anisotropy. The values average to 0.52 in good agreement with single donors measurements[S3].

Finally, we discuss the dimensions of the donor's envelope obtained from the fits with respect to the dimension of the Fourier filter. As shown in equations S1 and S2, the filters have a variance equal to $f_r(2\pi/a_0)$ (respectively $f_t(2\pi/a_0)$) in the longitudinal (transverse) direction. Back to real-space, this is obviously equivalent to a characteristic dimension $a_0/(2\pi f_r)$ in the longitudinal direction, which must be compared to the longitudinal envelope radii b, and to $a_0/(2\pi f_t)$ in the transverse direction to be compared with a. We consider here the values which correspond to f_r=0.10$(2\pi/a_0)$, as they are the ones presented in the main text. Over 16 values (2 valleys, 2 pairs, 2 donors/pair, experimental and theoretical images), the small envelope radii b range from 1.14 to 1.85 nm, with an average of 1.52 nm, which is much larger than the filter longitudinal dimension equal to 0.84 nm. Likewise, the large envelope radii b range from 2.34 to 3.48 nm, with an average of 2.96 nm, which is also much larger than the filter's transverse dimension equal to 1.23 nm. This demonstrates the relevance of our fitting procedure to accurately extract not only the phase differences but also the envelope parts of the valley images discussed throughout the manuscript. Importantly, the overlap between the envelopes can be confidently discussed, to further prove the robustness of the valley interference in these regions, as well as the anisotropy of the envelope longitudinal and transverse dimensions. These are the two core ingredients of the effective mass model used to discuss their impact on the exchange interaction.

Supplementary Note 2 - Spectroscopy of exchange-coupled donors

We give in Fig. S7 the spectroscopic data measured for pair #1 of the main text. Fig. S7b shows the differential conductance vs U along a cut though the two donors. The different charge state transitions can be identified as the main resonance peaks occurring around -0.5V, -0.8V and -1.15V (black arrows in Fig. S7b-c), for the 0e/1e, 1e/2e and 2e/3e, respectively, with the neutral 1e/2e state transition occurring close to the flat-band condition (zero electric field) at -0.8V because of tip-induced band bending[S1,S9,S10]. We also note that the 0e/1e transition is mainly located on the bottom donor from Fig. S7a. This might be due to a combination of stray electric field from the environment as well as electric field created by the tip at this repulsive bias value.

As mentioned, we do not expect the local tip-induced stray field to distort the 2e image because this image is taken at a bias close to the flat-band condition, i.e. zero electric field. More importantly, we expect the valley phase to be robust against electric fields from the tip, because the valley frequency is not sensitive to electric field and, even in the presence of a moderate Stark shift, the phase is always pinned to the donor's ion position.

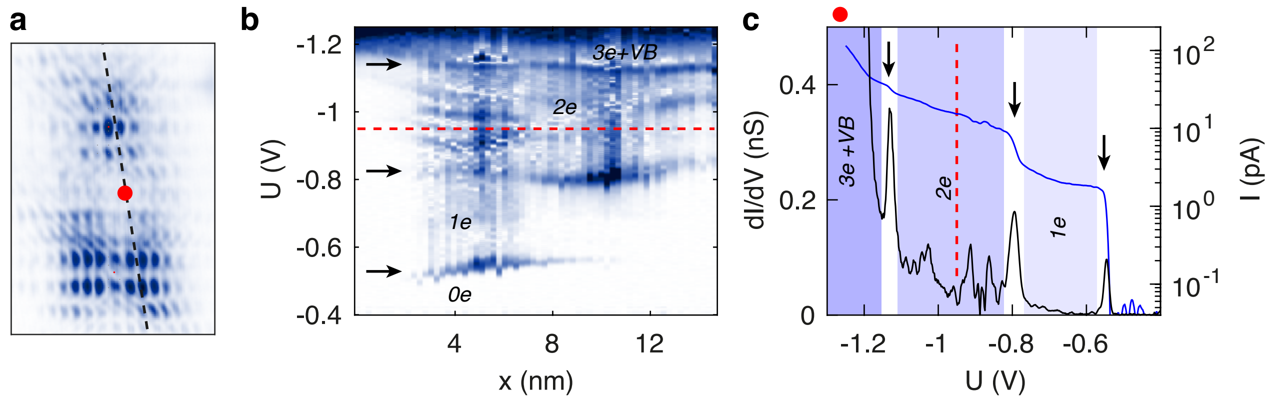

FIG. S7: **Spectroscopy data for the pair presented in the main text. a,** STM image of the pair discussed in the main text. **b,** Map of the differential conductance plotted vs bias voltage taken along the black dotted line shown in a. The different charge states (0e, 1e, 2e and 3e) can be identified from the main differential conductance peaks around, respectively, -0.5V, -0.8V and -1.15V, indicated by the black arrows. The red doted line indicates the bias voltage at which the STM image was taken, above the 1e/2e transition. **c,** Differential conductance plotted vs bias voltage taken at the red spot shown in a. The different charge state transitions can clearly be identified (black arrows).

Supplementary Note 3 - Valley interference, STM placed donors and exchange analysis

Heitler-London exchange for two donors in silicon.

In this section we derive the expression of the exchange interaction energy between two donors in the Heitler-London (HL) regime[S11]. For convenience we start from a single k-point, A1-like donor ground state:

$$\begin{aligned} \psi(\vec{r}) &= \sum_{\mu=1}^{6} \alpha_\mu F_\mu(\vec{r})\phi_\mu(\vec{r}) \\ with \quad F_{\pm z}(\vec{r}) &= \frac{1}{\sqrt{\pi a^2 b}} e^{-\sqrt{\frac{(x^2+y^2)}{a^2}+\frac{z^2}{b^2}}} \quad \text{anisotropic} \quad \text{envelope} \\ and \quad \phi_\mu(\vec{r}) &= u_\mu(\vec{r}) e^{i\vec{k_\mu}\cdot\vec{r}} \quad \text{Bloch} \quad \text{functions} \end{aligned} \tag{S12}$$

The α_μ represent the valley population distribution among the 6 valleys $\pm x$, $\pm y$ and $\pm z$ and are assumed to be real numbers. a and b represent the transverse and longitudinal donor envelope radii, respectively. The average ratio b/a=0.52 obtained in this work is in good agreement with reported values for single donors[S3], the value a is discussed below in the context of atomistic calculations of the exchange interaction.

The HL-exchange interaction energy is defined as:

$$\begin{aligned} J(\vec{R}) &= \iint d\vec{r}_1 d\vec{r}_2 \psi^*(\vec{r_1})\psi^*(\vec{r_2}-\vec{R}) \frac{e^2}{\epsilon|\vec{r_1}-\vec{r_2}|} \psi(\vec{r_1}-\vec{R})\psi(\vec{r_2}) \\ &= \iint d\vec{r}_1 d\vec{r}_2 \Big(\sum_\mu \alpha_\mu F^*_\mu(\vec{r_1})\phi^*_\mu(\vec{r_1})\Big)\Big(\sum_\nu \alpha_\nu F^*_\nu(\vec{r_2}-\vec{R})\phi^*_\nu(\vec{r_2}-\vec{R})\Big) \frac{e^2}{\epsilon|\vec{r_1}-\vec{r_2}|} \times \\ &\quad \Big(\sum_{\mu'} \alpha_{\mu'} F_{\mu'}(\vec{r_1}-\vec{R})\phi_{\mu'}(\vec{r_1}-\vec{R})\Big)\Big(\sum_{\nu'} \alpha_{\nu'} F^*_{\nu'}(\vec{r_2})\phi^*_{\nu'}(\vec{r_2})\Big) \\ &= \iint d\vec{r}_1 d\vec{r}_2 \sum_{\mu\nu\mu'\nu'} \alpha_\mu\alpha_\nu\alpha_{\mu'}\alpha_{\nu'} F_\mu(\vec{r_1})^* F_\nu(\vec{r_2}-\vec{R})^* \frac{e^2}{\epsilon|\vec{r_1}-\vec{r_2}|} F_{\mu'}(\vec{r_1}-\vec{R})F_{\nu'}(\vec{r_2}) \times \\ &\quad \phi_\mu(\vec{r_1})^*\phi_\nu(\vec{r_2}-\vec{R})^*\phi_{\mu'}(\vec{r_1}-\vec{R})\phi_{\nu'}(\vec{r_2}) \end{aligned} \tag{S13}$$

We define the envelope part $\tilde{j}_{\mu\nu\mu'\nu'} = F^*_\mu(\vec{r_1})F^*_\nu(\vec{r_2}-\vec{R})\frac{e^2}{\epsilon|\vec{r_1}-\vec{r_2}|}F_{\mu'}(\vec{r_1}-\vec{R})F_{\nu'}(\vec{r_2})$. We develop the Bloch function parts using:

$$u_\mu(\vec{r}) = \sum_{\vec{K}} c^\mu_K e^{i\vec{K}\cdot\vec{r}} \quad with \quad \sum_{\vec{K}} |c^\mu_K|^2 = 1 \tag{S14}$$

We then obtain:

$$\begin{aligned} J(\vec{R}) = \sum_{\mu\nu\mu'\nu'} \alpha_\mu\alpha_\nu\alpha_{\mu'}\alpha_{\nu'} \iint d\vec{r}_1 d\vec{r}_2 \tilde{j}_{\mu\nu\mu'\nu'} e^{i(\vec{k_{\mu'}}-\vec{k_\mu})\cdot\vec{r_1}} e^{i(\vec{k_{\nu'}}-\vec{k_\nu})\cdot\vec{r_2}} e^{i(\vec{k_\nu}-\vec{k_{\mu'}})\cdot\vec{R}} \times \\ \sum_{\vec{K}_1..\vec{K}_4} c^{\mu*}_{\vec{K}_1} c^{\nu*}_{\vec{K}_2} c^{\mu'}_{\vec{K}_3} c^{\nu'}_{\vec{K}_4} e^{-i\vec{K}_1\cdot\vec{r_1}} e^{-i\vec{K}_2\cdot(\vec{r_2}-\vec{R})} e^{i\vec{K}_3\cdot(\vec{r_1}-\vec{R})} e^{i\vec{K}_4\cdot\vec{r_2}} \end{aligned} \tag{S15}$$

A few assumptions are then made to obtain the final expression. First we neglect the fast oscillating terms in (S9) of the form $e^{i(\vec{k_{\mu'}}-\vec{k_\mu})\cdot\vec{r_1}}$ with $\mu' \neq \mu$ and likewise $e^{i(\vec{k_{\nu'}}-\vec{k_\nu})\cdot\vec{r_2}}$ with $\nu' \neq \nu$, which are called the inter-valley exchange terms as the electrons change valley and site during exchange. These fast oscillating terms would integrate to zero for inter-donor distances larger than the envelope radius[S8,S11] which we consider in this work. Therefore, we neglect them in the following and only consider intra-valley processes. Hence $\mu = \mu' \rightarrow \mu$ for electron 1 and $\nu = \nu' \rightarrow \nu$ for electron 2. Assuming $\alpha_\mu = \alpha_{\mu'}$ and $\alpha_\nu = \alpha_{\nu'}$ the expression for the exchange becomes:

$$J(\vec{R}) = \sum_{\mu\nu} \alpha_\mu^2 \alpha_\nu^2 \iint d\vec{r_1} d\vec{r_2} \tilde{j}_{\mu\nu} e^{i(\vec{k_\nu}-\vec{k_\mu})\cdot\vec{R}} \sum_{\vec{K_1}..\vec{K_4}} c^{\mu*}_{\vec{K_1}} c^{\nu*}_{\vec{K_2}} c^{\mu}_{\vec{K_3}} c^{\nu}_{\vec{K_4}} e^{i(\vec{K_3}-\vec{K_1})\cdot\vec{r_1}} e^{i(\vec{K_4}-\vec{K_2})\cdot\vec{r_2}} e^{i(\vec{K_2}-\vec{K_3})\cdot\vec{R}}$$
$$= \sum_{\mu\nu} \alpha_\mu^2 \alpha_\nu^2 \iint d\vec{r_1} d\vec{r_2} \tilde{j}_{\mu\nu} e^{i(\vec{k_\nu}-\vec{k_\mu})\cdot\vec{R}} \sum_{\vec{K_1}\vec{K_2}} |c^{\mu}_{\vec{K_1}}|^2 |c^{\nu}_{\vec{K_2}}|^2 e^{i(\vec{K_1}-\vec{K_2})\cdot\vec{R}} \tag{S16}$$

$\vec{K_1} - \vec{K_2}$ is a reciprocal lattice vector and $\vec{R}$ is a lattice vector for substitutional donors, thus $e^{i(\vec{K_1}-\vec{K_2})\cdot\vec{R}} = 1$. Using the normalisation condition for the Bloch states we finally obtain:

$$J(\vec{R}) = \sum_{\mu\nu} J_{\mu\nu} = \sum_{\mu\nu} \alpha_\mu^2 \alpha_\nu^2 \, j_{\mu\nu}(\vec{R}) \, \cos\Big((\vec{k_\mu} - \vec{k_\nu}) \cdot \vec{R}\Big)$$
$$with \quad j_{\mu\nu}(\vec{R}) = \iint d\vec{r_1} d\vec{r_2} \tilde{j}_{\mu\nu}(\vec{R}) = \iint d\vec{r_1} d\vec{r_2} F^*_\mu(\vec{r_1}) F^*_\nu(\vec{r_2} - \vec{R}) \frac{e^2}{\epsilon|\vec{r_1} - \vec{r_2}|} F_\mu(\vec{r_1} - \vec{R}) F_\nu(\vec{r_2}) \tag{S17}$$

similar to ref[S11,S12].

Phenomenological effective mass model

This section describes how we constructed the phenomenological effective-mass model (P-EM). We first start from the general exchange equation (S11). For the bulk donor devices fabricated by STM lithography we use constant $\alpha_\mu = 1/\sqrt{6}$, hence taking the valley population weights out of the equation, as we will only be interested in exchange variations later on. It reduces the exchange expression to only a sum of envelope terms modulated by valley interference:

$$J^{PEM}(\vec{R}) = \sum_{\mu,\nu=1}^{6} j_{\mu\nu}(\vec{R}) \, \cos\Big((\vec{k_\mu} - \vec{k_\nu}) \cdot \vec{R}\Big) \tag{S18}$$

The terms $(\vec{k_\mu} - \vec{k_\nu}) \cdot \vec{R}$ can be rewritten as $\Delta\phi_{\mu\nu}$ as used in the main text with $\Delta\phi_{\mu\nu} = \text{sign}(\mu)\Delta\phi_\mu - \text{sign}(\nu)\Delta\phi_\nu$ which makes the link with the valley phase differences experimentally measured. The envelope terms can be simplified assuming $F_\mu(\vec{r_1})F_\nu(\vec{r_2}) \sim F_\mu(\vec{r_1})F_\nu(\vec{r_2})\delta(\vec{r_1} - \vec{r_2})$ because of the exponential nature of the orbitals. This leads to:

$$j_{\mu\nu}(\vec{R}) = \iint d\vec{r_1} d\vec{r_2} F^*_\mu(\vec{r_1}) F^*_\nu(\vec{r_2} - \vec{R}) \frac{e^2}{\epsilon|\vec{r_1} - \vec{r_2}|} F_\mu(\vec{r_1} - \vec{R}) F_\nu(\vec{r_2})$$
$$\sim \int d\vec{r_2} F^*_\mu(\vec{r_2} + \vec{R}) F^*_\nu(\vec{r_2} - \vec{R}) \frac{e^2}{\epsilon|\vec{R}|} F_\mu(\vec{r_2}) F_\nu(\vec{r_2})$$
$$\sim \int d\vec{r_2} F^*_\mu(\vec{r_2} + \vec{R}) F^*_\nu(\vec{R} - \vec{r_2}) \frac{e^2}{\epsilon|\vec{R}|} F_\mu(\vec{r_2}) F_\nu(\vec{r_2})$$
$$\sim \frac{1}{|\vec{R}|} F_\mu(\vec{R}) F_\nu(\vec{R}) F_\mu(\vec{0}) F_\nu(\vec{0}) = j^{PEM}_{\mu\nu}(\vec{R}) \tag{S19}$$

The products $F_\mu(\vec{R})F_\mu(\vec{0})$ (respectively $F_\nu(\vec{R})F_\nu(\vec{0})$) reflect the 3D exchange integrals for the electron exchanged in valley μ (respectively ν) between the two donors, and still contain the anisotropy and exponential nature of the envelope integrals. For instance, as discussed in the main text, considering two donors along [100] and $\vec{R} = na_0\vec{x}$:

$$F_x(na_0\vec{x})F_x(\vec{0}) \ll F_z(na_0\vec{x})F_z(\vec{0}) \quad \Rightarrow \quad j^{PEM}_{xz}(na_0\vec{x}) \ll j^{PEM}_{zz}(na_0\vec{x}) \tag{S20}$$

An illustrative case - [110]

The P-EM model developed above shows a complex 3D interplay between envelope anisotropic weights modulated by valley phase differences $\Delta\phi_\mu$. In order to illustrate their specific role, we can temporarily ignore any dopant misplacement and focus on the [110] direction only. Along this direction, we easily find from a symmetry argument $\Delta\phi_x = \Delta\phi_y = \Delta\phi_{[110]}$ and $\Delta\phi_z = 0$. The phase difference $\Delta\phi_{[110]}$ is plotted in Fig. S8a vs the inter-donor distance. A clear qualitative correlation arises between $\Delta\phi_{[110]}$ (wrapped between 0 and π) and the exchange energy computed from FCI, plotted in Fig. S8b: destructively interfering positions, i.e. $\Delta\phi_{[110]}$ close to π, match local minima in the exchange, with local variations of one to two orders of magnitude. This remains valid on a large range of distance, from a few nanometers where the exchange energy compares with the valley-orbit splitting and results in a triplet orbital mixing[S7,S13], to a larger distance limit d > 8 nm where the Heitler-London (HL) theory accurately describes the molecular state[S11,S14]. The anisotropic envelope plays a role in two ways. First it results in the overall exponential decay of the exchange magnitude with a characteristic length matching the large donor envelope radius a. Secondly the anisotropy results in the valley-induced exchange variations to be damped at large distances because of the dominance of the j_{zz} terms (constructive along [110]) over the j_{xz} and j_{yz} oscillating at $\Delta\phi_{[110]}$.

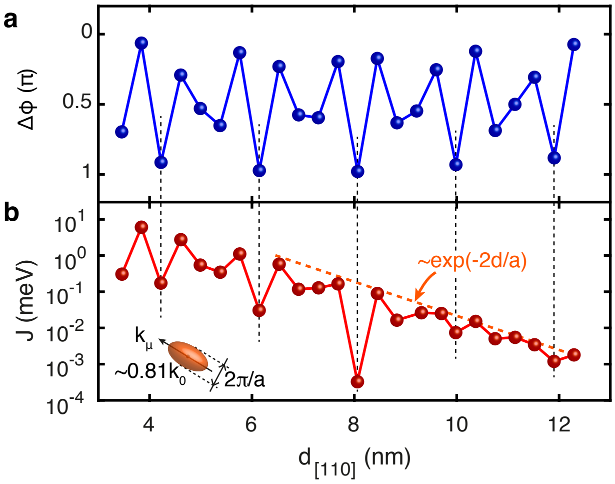

FIG. S8: **Valley interference and exchange interaction. a,** Theoretical phase difference along [110], where $\Delta\phi_x = \Delta\phi_y = \Delta\phi$, wrapped between 0 and π, plotted vs. inter-donor distance along [110]. **b,** Corresponding exchange value obtained from TB/FCI calculations. $\Delta\phi$ and J present a similar oscillating behaviour, with local J minima (resp. maxima), corresponding to valleys being out-of-phase (resp. in-phase), i.e. $\Delta\phi$ close to π (resp. 0), as indicated by the vertical dashed lines. The red dashed line indicates the exponential decay due the overlap of the wavefunctions envelope parts only, with a characteristic decay length given by the large donor envelope radius a.

STM analysis - two-donor position configurations

We detail here the different donor position configurations when they are meant to be placed along [100] or [110]. The 6 possible positions for each donor results in 36 possibilities for the placement of the two donors, some of them being equivalent. A target along [100] (Fig. S9a) results in 12 non-equivalent donor-donor position configurations with associated occurrence numbers (adding up to 36) represented in Fig. S9b, with the convention of only moving P_2 (P_1 fixed). Using the same convention, a target along [110] results in 10 non-equivalent configurations represented in Fig. S9d, also with associated occurrence numbers.

The P-EM model only uses four parameters: the position of the conduction band minimum k_μ, the envelope radii a and b, which have been measured experimentally, and a normalisation constant $\mathcal{N}$ which can be ignored as we focus on exchange variations and not absolute values. In order to check the relevance of this model we compared it to existing calculations. Gamble *et al.* published a complete set of tunnel coupling t calculations in 3D[S15]. We considered all the points in an in-plane slice around a target distance of 12 nm as shown in Fig. S10a, squared them to obtain a quantity proportional to exchange (simply assuming $J \sim t^2/E_C$ in the HL limit, where E_C is the charging

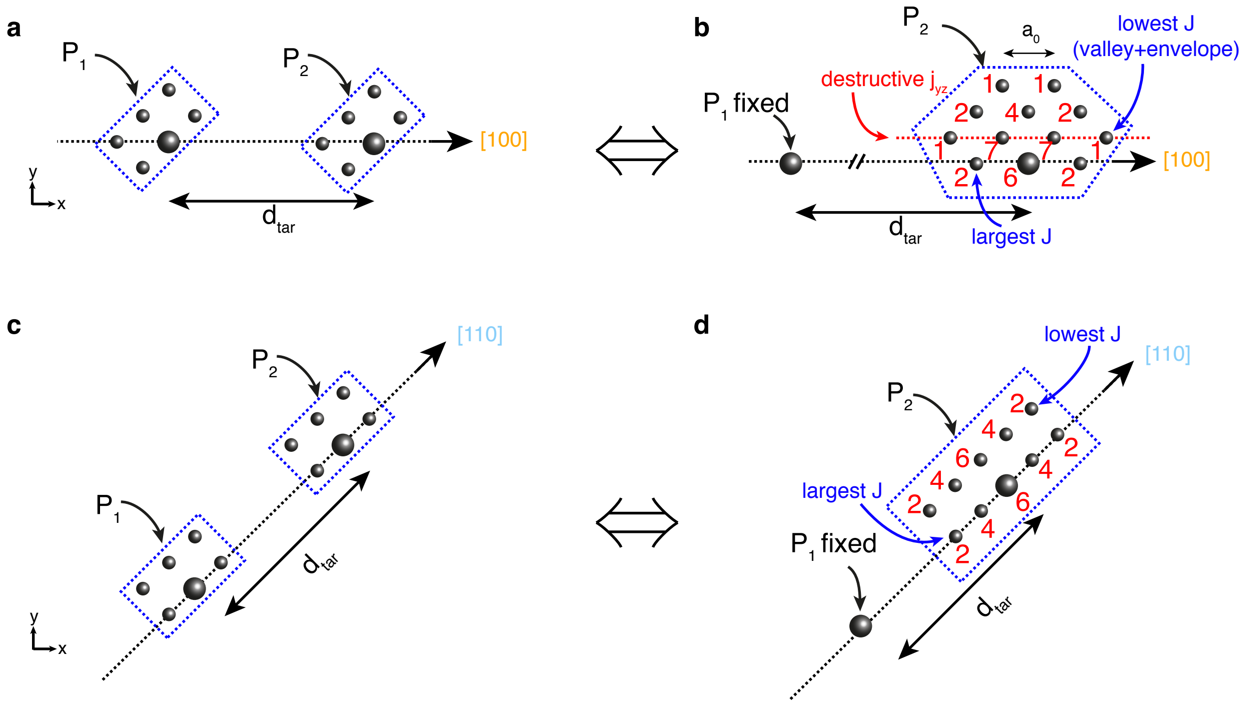

FIG. S9: **Statistical analysis of the two-donor position configurations. a,** Two donors placed along [100], there is 6 possible sites for each of them, i.e. 36 possibilities in total. **b,** For the purpose of the statistical analysis we can fix the position of P_1 and work out the 12 possible, non-equivalent, positions for P_2 as well as their occurrence number (assuming a random distribution of each donor across their 6 possible sites), shown in red, adding up to 36. The destructive positions off by $a_0/2$ along the y-axis resulting in low exchange values (see main text) add up to 16 out the 36 possibilities, i.e. 44% chance to be hit. **c,** Two donors placed along [110], there is 6 possible sites for each of them, i.e. 36 possibilities in total. **d,** There are 10 resulting non-equivalent positions for P_2, assuming P_1 fixed.

energy set as constant), normalised them and fitted them using the PEM model. Fig. S10b shows an exceptional level of agreement, with notably $k_\mu = 0.84k_0$ as used in this reference. Furthermore we empirically calibrated this P-EM model to Heitler-London exchange calculations along [110] based on a tight-binding basis, shown in fig. S10c. The table shown in Fig. S10d gives the resulting fitting coefficients obtained for these two fits, with a good agreement between the two theories. The difference in k_μ (0.84 against 0.81 in our work) accounts for why the exchange minima are getting out-of-phase with each other at such distances, as observed in ref[S15]. The value $a = 2.8\,\text{nm}$ is used in the main text and in this Supplementary Information for any P-EM calculation.

Long distance limit

We can calculate a set of exchange values using the P-EM model for different target distances along both [100] and [110], and extract the minimum and maximum values, respectively J_{min} and J_{max} for each set. We plot in Fig. S11a the resulting exchange variations, i.e. $r_{[100]} = J_{min}/J_{max}$ along [100], and likewise $r_{[110]}$ along [110], as a function of d_{tar}. Along [100] the large variations of more than two orders of magnitude are invariably present due to the destructive locations which are one lattice site off the [100] axis. We can determine the asymptotic limit of $r^{\infty}_{[100]}$, knowing the donor locations leading to the largest and lowest exchange values (see Fig. S9), and only considering the j_{zz}, j_{yz} and j_{yy} terms (representing 16 out of the 36 terms in total), defining $d_{tar} = Na_0$:

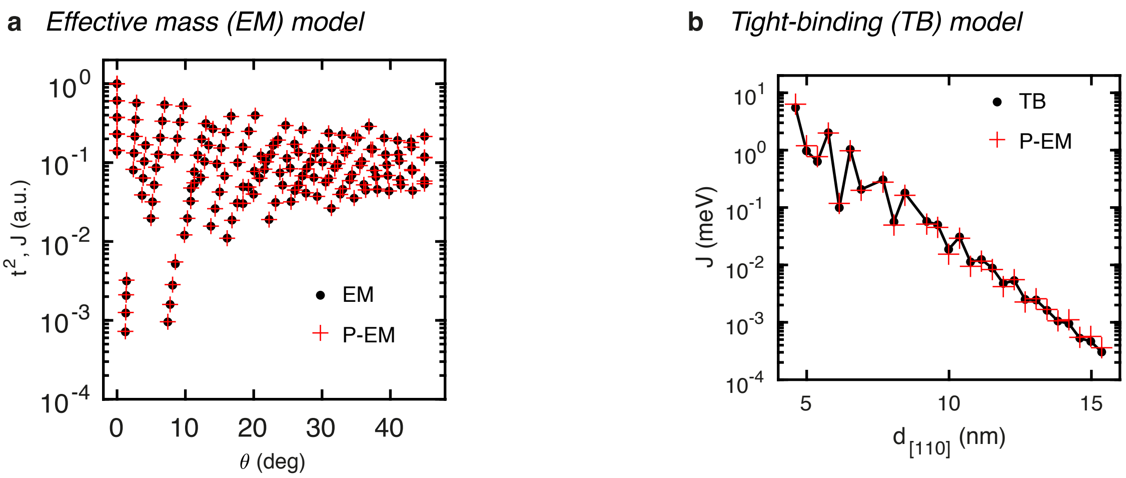

c

	Effective mass Gamble	HL-TB
k_μ	$(0.8402 \pm 0.0001)*k_0$	$(0.814 \pm 0.002)*k_0$
a (nm)	2.46 ± 0.01	2.82 ± 0.06
b/a	0.482 ± 0.001	0.50 ± 0.01

FIG. S10: **Comparison and fit of the P-EM model.** **a,** Fit to the square of the tunnel coupling values obtained in Gamble *et al.*[S15], for 148 in-plane positions located close to a target distance of 12 nm, between [100] and [110]. **b,** Fit to the tight-binding calculations developed in this work along [110]. **c,** Table summarising the fitting parameters obtained in each case. Equations (S18) and (S19) were used to fit both cases, along with a normalisation constant.

$$r_{[100]} = \frac{J_{min}}{J_{max}} \sim \frac{(6 + 8\cos\left(\frac{2\pi}{2}0.81\right) + 2\cos(2\pi 0.81))\exp\left(-2a_0/a * \sqrt{(N+1.5)^2 + (0.5)^2}\right)}{16\exp(-2(N-1)a_0/a)}$$
$$\xrightarrow[N\to\infty]{} r^{\infty}_{[100]} = \frac{3 + 4\cos\left(\frac{2\pi}{2}0.81\right) + \cos(2\pi 0.81)}{8}\exp(-5a_0/a) \sim 2.8\times 10^{-3} \tag{S21}$$

assuming a=2.8 nm. We have described in the main text the dominance of the j_{zz} terms for donors placed around the [110] direction over the j_{yz} and j_{xz} terms, with a ratio j_{zz}/j_{yz} growing exponentially with distance. In-plane valley-induced variations are hence washed out by the dominance of the j_{zz} terms in the long distance limit. For target distances beyond 12 nm the variations approach an envelope limit, only set by the distance difference between the shortest and longest inter-donor distances, i.e:

$$r_{[110]} = \frac{J_{min}}{J_{max}} \sim \frac{\exp\left(-2a_0/a * \sqrt{(N+1.5)^2 + (N+0.5)^2}\right)}{\exp\left(-2\sqrt{2}(N-1)a_0/a\right)}$$
$$\xrightarrow[N\to\infty]{} r^{\infty}_{[110]} = \exp\left(-4\sqrt{2}a_0/a\right) \sim 0.33 \tag{S22}$$

The exchange variation analysis we have developed here can be extended to the tunnel coupling, using the relationship $J = 4t^2/U$ valid in the Heisenberg regime, i.e. in the weak coupling limit $t \ll U$ and $t \ll \Delta_{VO}$ (where Δ_{VO} is the valley-orbit splitting of the order of 10 meV). This limit corresponds to inter-donor distances larger than 5 nm[S7,S15,S16]. The tunnel coupling variations can easily be deduced from the exchange coupling variations since $t_{min}/t_{max} = \sqrt{J_{min}/J_{max}}$, plotted in Fig. S11b vs the target distance along [110] or [100]. We found the tunnel coupling variations are lower than a factor of 5 for any target distance along [110] beyond 5 nm, as mentioned in the main text.

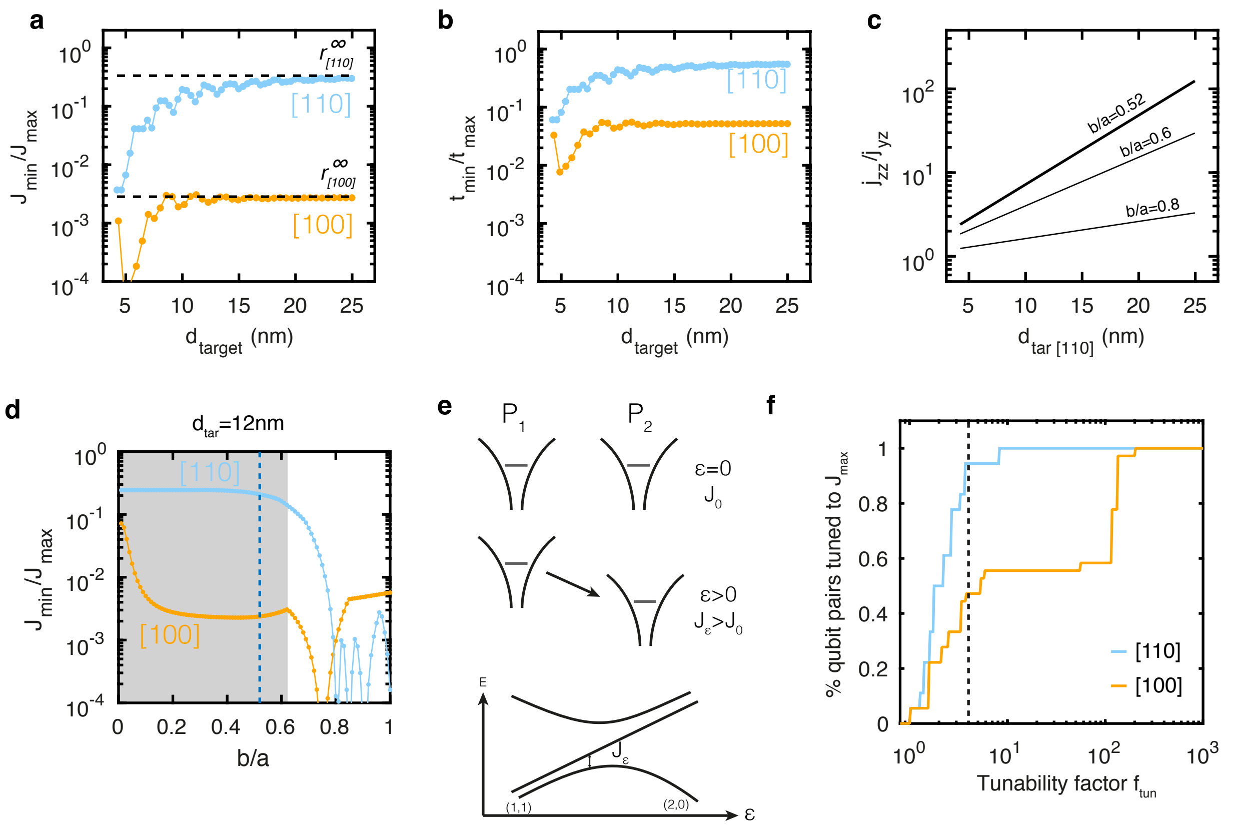

FIG. S11: **Envelope-limited exchange variations and exchange tunability. a,** Plot of exchange variations J_{min}/J_{max} and of their asymptotic limit at large d_{tar} of the exchange values obtained from STM dopant placement vs target distances along [100] (orange) and [110] (blue). The variations along [110] reach the envelope limit of a factor 1/0.33, i.e. variations due to the change in inter-donor distance from donor misplacement, while the [100] consistently show more than two orders of magnitude of variations and due to the presence of destructive positions off the [100] axis by $a_0/2$ along y. **b,** Plot of tunnel coupling variations t_{min}/t_{max} vs target distances along [100] (orange) and [110] (blue), in the Heisenberg limit valid for inter-donor distances larger than 5 nm. The variations along [110] are consistently lower than a factor of 5 for this distance range.**c** Ratio j_{zz}/j_{yz} plotted vs d_{tar} along [110] for different anisotropy ratios b/a. A ratio below 0.6 protects the exchange interaction against in-plane valley interference, i.e. $\Delta\phi_y$ and $\Delta\phi_x$. **d** Exchange variations plotted vs b/a for d_{tar}=12 nm for both [110] and [100] target orientation. The shaded area corresponds to the region where the exchange along [110] is protected against x and y-valley interference, i.e. for b/a<0.6. **e,** Exchange can be tuned using energy detuning between the two atoms, inducing a (2,0) component in the charge configuration with a larger exchange than the (1,1) configuration. **f,** Percentage of qubits tuned to the maximum value as a function of a tunability factor.

The asymptotic regime is approached for target distances beyond 12 nm, for both exchange and tunnel coupling, as shown in Fig. S11a and b. This a relevant distance range where the exchange coupling has been predicted to be tunable by more than a factor of 5 using electric field detuning[S14]. This tuning scheme is based on inducing in the ground state a (2,0) charge component (where the two electrons are on the same site), which has a much larger exchange energy than the pure (1,1) state obtained at zero detuning (i.e. zero electric field), as schematised in Fig. S11b. We plot in Fig. S11c the percentage of qubits which could be tuned to the same value, i.e. the highest one as this tuning scheme can only increase the exchange, as a function of a tuning factor f_{tun}. There are 9 steps for the [110] curve corresponding to the 9 non-equivalent configurations lower than the maximum one, 11 for the [100] curve. A step occurs at a given f_{tun} when a configuration of exchange J reaches the condition $J * f_{tun} = J_{max}$. Each step height reflects the occurrence number of the corresponding configuration, discussed in Fig. S9. Notably, along [100] the positions leading to destructive j_{yz} and low J values count for 16 out of the 36 possibilities, i.e. almost 50%, hence large tuning factors (more than 100) are required to tune all the exchange values beyond this threshold. However, along [110] a realistic tuning factor of 4[S14] is sufficient to tune more than 94% of the qubit pairs tuned to the same exchange value, reaching the minimum working qubit yield for quantum error correction to be in principle implemented[S17].

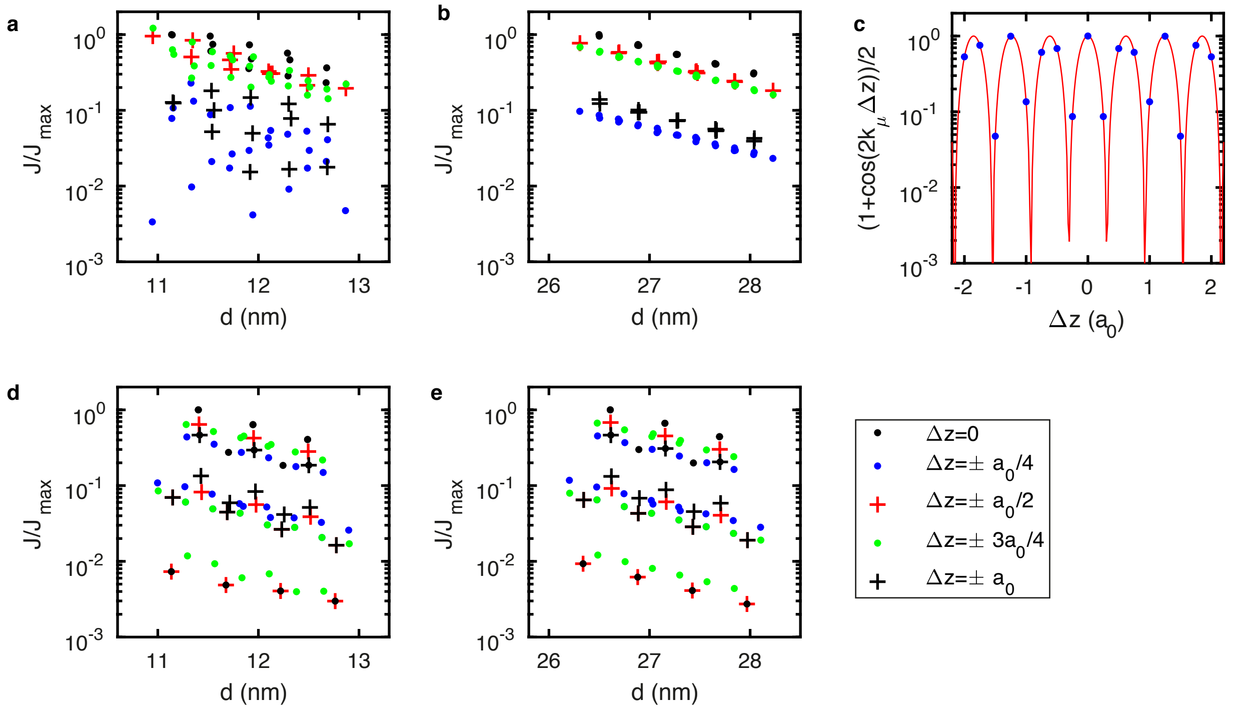

FIG. S12: **Exchange variation analysis including out-of-plane interference. a,** Plot of the exchange values for inter-donor positions found within 1 nm in-plane circle centred on 12 nm target distance, and within $\pm 1a_0$. An arrangement emerges as a function of the z-plane difference between the two donors. **b,** Same for a target distance of 27 nm. The arrangement is fully set as the exchange only rely on the z-valley interference and an envelope function with $J \sim (1+\cos(2\Delta\phi_z))e^{-2d/a}$. **b,** Plot of the z-valley interference induced exchange variations ($(1+\cos(2\Delta\phi_z))/2$, red line) for the available inter-atomic plane between the donors (blue spots). **c,** Same as **a,** for a 12 nm target distance along [100]. A complex arrangement can be seen, function of $\Delta\phi_y$ and $\Delta\phi_z$ as the j_{yy}, j_{yz} and j_{zz} terms are degenerate. No inter-atomic plane shows a larger exchange variations than that of the in-plane ones discussed in the main tex, which also exist for the $a_0/2$ z-plane difference by symmetry. **d,** Same as **c,** for a 27 nm target distance.

z-valley interference and exchange

An important finding of the manuscript is the protection against x and y-valley interference along [110]. Here we develop the influence of the z-valley interference when the donors are not in the same plane. We have computed the exchange variations according to the P-EM model for all the positions included within a 1 nm in-plane neighbourhood and within ± 1 monolayer, i.e. $\pm a_0$, for in-plane target distances of 12 nm and 27 nm along [100] or [110]. As it is mentioned in the main text, the dependence of the exchange interaction with the z-valley interference along [110] is straightforward following the dominance of the j_{zz} terms. In the asymptotic limit, the exchange can be reduced to simply relate to the z-valley phase only with $J \sim (1+\cos(2\Delta\phi_z))e^{-2d/a}$. This allows to predict exchange variations including possible donor misplacement in the z-direction. Since $\Delta\phi_z$ is the only relevant valley phase difference, the exchange variations will be arranged as a function of the depth difference, i.e. atomic planes, between the donors. This was evidenced and discussed in Fig.7 of the main text. This arrangement already emerges for a target distance of 12 nm as seen in Fig. S12a, with an exchange reduced for donor pairs with either a $a_0/4$ or a_0 z-coordinate difference, and has become asymptotic for a 27 nm target distance as shown in in Fig. S12b. The worst case is for the donors to end up at $a_0/4$ depth difference from each other, resulting in variations of $2\Delta J^{\infty}_{[110]}/(1+\cos(0.81\pi)) \sim 41.6$. We plot in Fig. S12c the exchange variations due to the z-valley interference for a z range within $\pm 2a_0$, aligning the possible lattice positions (blue spots) on the resulting variations given by $(1+\cos(2k_\mu\Delta z))/2$.

The exchange variations for target distances along the [100] axis are plotted in Fig. S12d and for 12 and 27 nm target distances respectively. There, an arrangement can also be seen, however it is more complex than the [110] case, as the degeneracy between the j_{yy}, j_{yz} and j_{zz} terms results in both $\Delta\phi_z$ and $\Delta\phi_y$ to be involved. This arrangement

is already well set up at 12 nm target distance because the dominance of these terms is even more pronounced than the dominance of j_{zz} along [110] (see Fig.6b of main text). Nevertheless, it appears that the worst case within $\pm 1a_0$ variations in z is already contained in the in-plane exchange variations discussed in the main text, which are also present in the $\Delta z = a_0/2$ plane by symmetry. This confirm the destructive character of these positions along [100] for the exchange, with the largest variations along [110] including out-of-plane variations being still about one order of magnitude less than the in-plane exchange variations along [100].

In-plane valley interference and exchange along [100] and [110] - Literature comparison.

In this section, we demonstrate that our results are fully consistent with previous work, and also allow to reconcile their apparent different conclusions. Reference[S11] points out to the existence of suppressed exchange values in the close neighbourhood of a target position, reference[S12] favours the [100] axis for donor placement because of the absence of exchange oscillations along this axis, and reference[S15] mentions the absence of a sizeable region where the exchange is stable. References[S11] and [S12] can be directly compared to our results since they are all based on HL formalism using effective mass wavefunctions, following eq. S12 with however different parameters summarised in Fig. S13a. We note that the 3 models have a very similar envelope ratio b/a but reference[S12] computed a much smaller major Bohr radius (0.9 nm against 2.5 nm). The damping of exchange oscillations purely along [100] is related to the ratio between the j_{xz} and the j_{zz} terms. Following the expressions S19 given above and assuming $x > 0$ and $b < a$, this ratio reads:

$$(j_{xz}/j_{zz})(x)_{[100]} \sim \exp\Big(-\frac{x}{a}(\frac{a}{b} - 1)\Big) \xrightarrow[x\to\infty]{} 0 \qquad \text{(S23)}$$

This expression makes clear that the x-valley interference impact on the exchange vanishes for a finite anisotropy $b < a$, and is also more pronounced at fixed inter-donor distance along [100] for small a[S12]. Crucially, damping the x-valley exchange oscillations also leaves a degeneracy between the j_{yy}, j_{yz} and the j_{zz} terms, from which result the suppressed exchange values in the close neighbourhood of [100] because of destructive y-valley interference (see main text and "long distance limit" section above). We show in Fig. S13c-d, the normalised exchange values (with the envelope part taken away in order to focus on the valley interference only) along [100] expected for each model. Both models show over a factor of 100 reduction in exchange for the destructive y-positions (see Fig. S13b), very consistent with both our models shown in Fig.7 of the main text. That is because it is a pure valley interference effect as long as the anisotropy is large enough to create the gap between the j_{xz} and the j_{zz} terms, which we show is largely achieved for these models for very moderate inter-donor distances above 5 nm.

Along [110], the ratio between the j_{xz} (or j_{yz} as $y = x$) and the j_{zz} terms along [110] reads:

$$(j_{xz}/j_{zz})(x)_{[100]} \sim \exp\left(-\frac{\sqrt{2}x}{a}(\sqrt{\frac{a^2+b^2}{2b}} - 1)\right) \xrightarrow[x\to\infty]{} 0 \qquad \text{(S24)}$$

Exchange oscillations are also damped along [110], however with a smaller rate compared to [100] since $\sqrt{a^2+b^2} < 2a$. This can visually be seen in Fig.6b of the main text as the gap between the j_{xz} and j_{zz} terms along [100] is larger than that of along [110]. Crucially, along [110] the envelope anisotropy damps both the x-valley and the y-valley oscillations, hence yielding a 2D exchange protection against valley interference, instead of the 1D protection (as x-valley interference are damped only) along [100]. This 2D protection shown in Fig.7d of the main text is well reproduced in Fig. S13e-f using the parameters found in reference[S12] and[S11], respectively, with also less than a factor 10 exchange variation. We also show in Fig. S13f-g that both the strong exchange reduction along [100] and the 2D protection along [110] also features in reference[S15] by simply squaring their tunnel coupling calculations, which is also apparent in Fig.S10a.

In summary, considering a crystallographic axis for exchange stability with no off-axis displacement makes the [100] direction appealing[S12] because of the faster decay of the x-valley interference impact on exchange, which is enhanced for smaller a values too. But the sensitivity to y- and z-valley interference along [100] remains, and can be extremely severe when dopants are misplaced from this axis. Instead, [110] results in a higher dimensional protection, which is suitable to the dopant placement accuracy provided by STM lithography. We have shown how considering

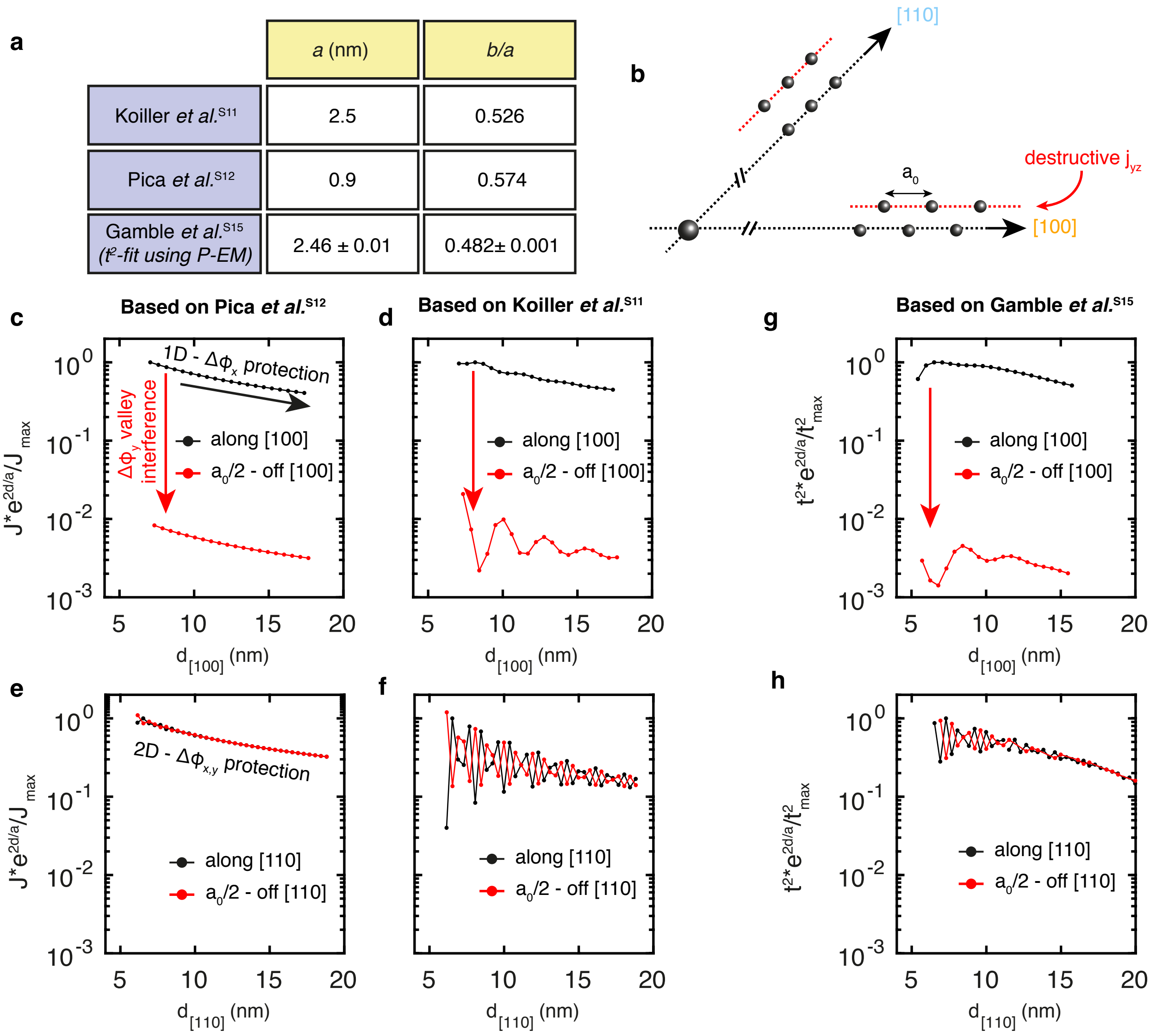

FIG. S13: **In-plane valley interference and exchange along [100] and [110] - Literature comparison. a,** Table summarising the effective mass wavefunctions parameters directly found in reference[S12] and[S11], and obtained from the fits of the calculations found in reference[S15] to our P-EM model. The envelope anisotropy b/a are similar but reference[S12] found a smaller major Bohr radius a compared to reference[S11] and our work. **b,** Dopant coordinates on and closest off the [100] and [110] axis. **c,** Normalised HL exchange values on and off the [100] axis, computed based on reference[S12] parameters. The exchange envelope decay $\sim \exp(-2d/a)$ was compensated, the remaining decay is due to the Coulomb potential. **d,** Same for using parameters based on [S11]. For both set of parameters, the exchange is suppressed by more than a factor 100 for the off-[100] positions, which is fully consistent with our results. **e-f,** Same for the [110], showing that the exchange is protected against in-plane position variations with less than a factor 10 variation, again consistent with our results. **f-g,** Normalised square of the tunnel coupling calculations directly taken from reference[S15]. The envelope decay was also compensated, taking for a the value obtained from the fit to the P-EM model (Fig S10c). The exchange suppression along [100] as well as the exchange protection along [100] are also very well reproduced.

the interplay between dopant placement, envelope anisotropy and valley interference has allowed to elucidate the origin of the different messages which have been delivered in the literature, and we demonstrate here the universality of our results across different references and models.

Supplementary Note 4 - Robust two-qubit gates using the exchange interaction

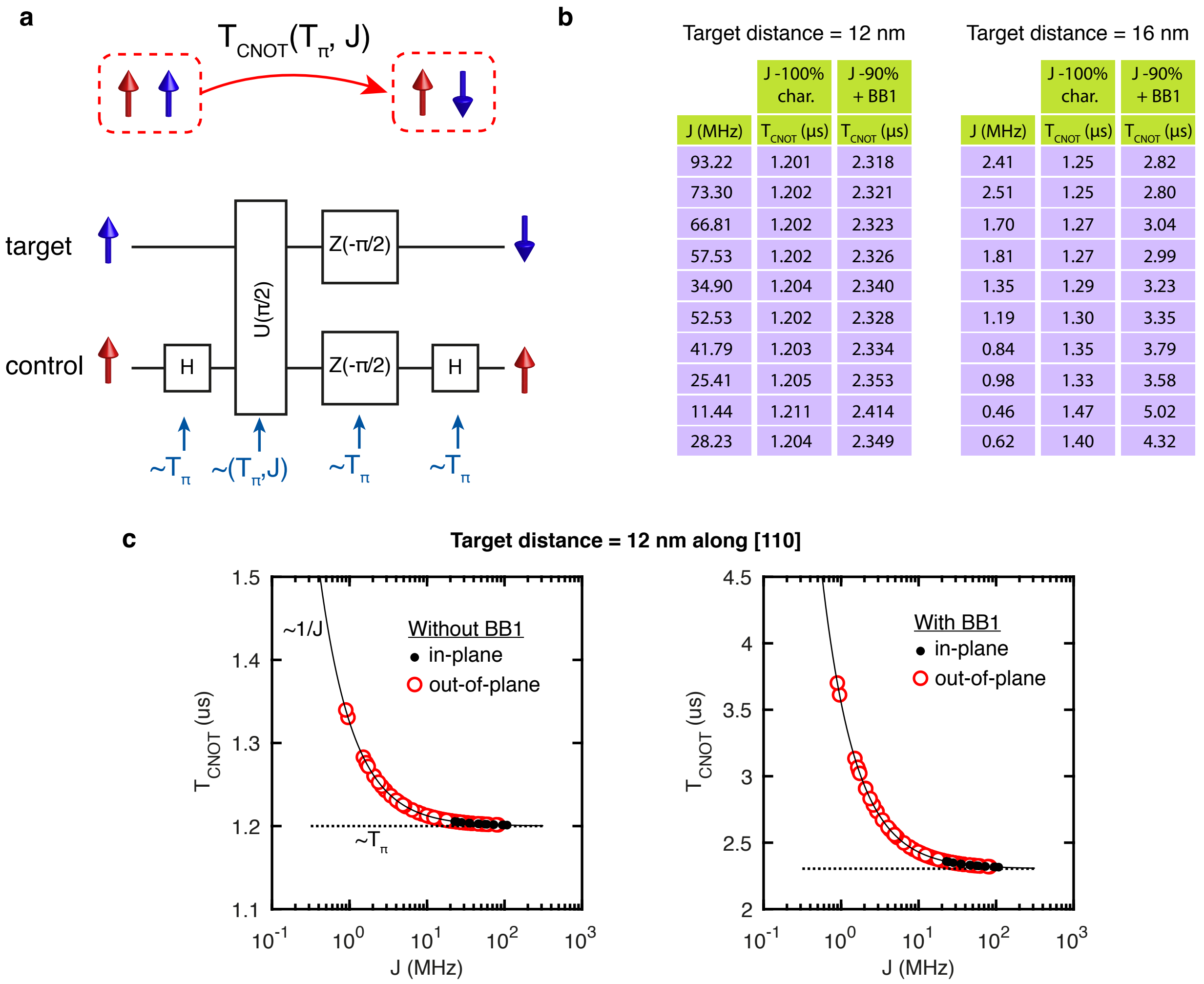

Target distance = 12 nm

J (MHz)	J -100% char. T_{CNOT} (μs)	J -90% + BB1 T_{CNOT} (μs)
93.22	1.201	2.318
73.30	1.202	2.321
66.81	1.202	2.323
57.53	1.202	2.326
34.90	1.204	2.340
52.53	1.202	2.328
41.79	1.203	2.334
25.41	1.205	2.353
11.44	1.211	2.414
28.23	1.204	2.349

Target distance = 16 nm

J (MHz)	J -100% char. T_{CNOT} (μs)	J -90% + BB1 T_{CNOT} (μs)
2.41	1.25	2.82
2.51	1.25	2.80
1.70	1.27	3.04
1.81	1.27	2.99
1.35	1.29	3.23
1.19	1.30	3.35
0.84	1.35	3.79
0.98	1.33	3.58
0.46	1.47	5.02
0.62	1.40	4.32

FIG. S14: **Robust CNOT gate based on exchange-coupled donors. a,**. A CNOT gate flips a target spin depending on the sign of a control spin. A composite sequence can achieve beyond 99.9% fidelity assuming only an exchange characterised to 10%. It is made of a combination of single qubit rotations, with characteristic time T_π, and of two-qubit rotations, with a characteristic time h/J. **b,** Tables of the calculated CNOT gate times for two target distances along [110], 12 nm (left) and 16 nm (right). The 12 nm histogram shows a very limited spread (1% without BB1, 4% with BB1) as the CNOT gate times are limited by T_π and hence not influenced by small variations in J. The exchange values obtained for a target distance of 16 nm are lower than the Rabi frequency $1/T_\pi$, hence the CNOT gate times are there limited by J and sensitive to exchange variations, accounting for the larger values and spread. It can be compensated by tuning the exchange values as presented in Fig. S11, bringing more than 94% of the exchange values (hence CNOT gate times) to the same values. **c,** Plot of the CNOT gate time values expected for a target distance of 12 nm, for both without (left) and with (right) the BB1 correction step, for both in-plane and out-of-plane dopant positions according to Fig. S12.

This section describes how a robust CNOT gate based on exchange interaction can be constructed following a sequence of single and two-qubit gates developed in[S18,S19]. As shown in Fig. S14a, this sequence consists of Hadamard gates, i.e. a single qubit $\pi-$rotation around the $\vec{x}+\vec{z}$ axis, $U(\pi/2)$ a two-qubit rotation and $Z(-\pi/2)$ single qubit $\pi/2$-rotation around the Z-axis. The calculated CNOT gate times are given in the tables shown in Fig. S14b, for two target distances 12 and 16 nm along [110]. As mentioned in the main text, for 12 nm the exchange values range between 93 and 11 MHz, i.e. all larger than the Rabi frequency, fixed at 3 MHz[S20]. This target distance represents a good compromise between benefiting from the [110] exchange stability scheme and from large

enough exchange values for the CNOT gate times to be mainly limited by T_π. The spread in CNOT gate time expected for the in-plane configurations is limited to 1% around a mean value of 1.2 μs. Considering out-of-plane configurations within 1 monolayer, the maximal CNOT gate time is 1.34 μs (see Fig. S14c). The BB1 sequence compensates for rotation errors if the exchange values are characterised to only 90% accuracy experimentally, to keep fidelities above 99.9%. In this case, in-plane CNOT gate times increased to an average value of 2.3 μs with a spread limited to 4%. The expected BB1 CNOT gate time for the out-of-plane configurations can reach up to 3.7 μs for the lowest exchange values, however we note that only 6 out of 82 computed configurations led to an operation time above 3 μs. This means that for this target distance, a factor of 100 variations in exchange results in less than a factor of 2 in the CNOT gate time. We performed similar calculations and analysis for two donors placed at 16 nm along [110]. For this target distance, the exchange values range between 2.4 and 0.5 MHz, which is comparable or lower than the Rabi frequency. As a consequence, the spread in exchange has more impact on the CNOT gate times, ranging now between 1.25 and 1.40 μs (up to 3.5 μs for out-of-plane configurations), and between 2.8 and 5.0 μs (up to 25.4 μs for out-of-plane configurations) if a BB1 step is involved. The in-plane spreads can be compensated by electrically tuning the exchange values as presented in the previous section, bringing more than 94% of the qubit pairs to the exact same exchange value with a realistic tuning factor of 4[S14].

[S1] J. Salfi, J. A. Mol, R. Rahman, G. Klimeck, M. Y. Simmons, L. C. L. Hollenberg, and S. Rogge. Spatially resolving valley quantum interference of a donor in silicon. *Nature Materials*, 13(6):605–610, 2014.

[S2] Muhammad Usman, Juanita Bocquel, Joe Salfi, Benoit Voisin, Archana Tankasala, Rajib Rahman, Michelle Y Simmons, Sven Rogge, and Lloyd C. L. Hollenberg. Spatial metrology of dopants in silicon with exact lattice site precision. *Nature Nanotechnology*, 11(June):1–19, 2016.

[S3] A. L. Saraiva, J. Salfi, J. Bocquel, B. Voisin, S. Rogge, Rodrigo B. Capaz, M. J. Calderón, and Belita Koiller. Donor wave functions in si gauged by stm images. *Phys. Rev. B*, 93:045303, Jan 2016.

[S4] Archana Tankasala, Joseph Salfi, Juanita Bocquel, Benoit Voisin, Muhammad Usman, Gerhard Klimeck, Michelle Y. Simmons, Lloyd C. L. Hollenberg, Sven Rogge, and Rajib Rahman. Two-electron states of a group-v donor in silicon from atomistic full configuration interactions. *Phys. Rev. B*, 97:195301, May 2018.

[S5] Massimo Rontani and Elisa Molinari. Imaging quasiparticle wave functions in quantum dots via tunneling spectroscopy. *Phys. Rev. B*, 71:233106, Jun 2005.

[S6] Rajib Rahman, Seung H Park, Gerhard Klimeck, and Lloyd C L Hollenberg. Stark tuning of the charge states of a two-donor molecule in silicon. *Nanotechnology*, 22(22):225202, apr 2011.

[S7] A L Saraiva, A Baena, M. J. Calderón, and Belita Koiller. Theory of one and two donors in silicon. *Journal of Physics: Condensed Matter*, 27(15):154208, 2015.

[S8] J. Salfi, J. A. Mol, R. Rahman, G. Klimeck, M. Y. Simmons, L. C. L. Hollenberg, and S. Rogge. Quantum simulation of the hubbard model with dopant atoms in silicon. *Nature Communications*, 7:11342–, April 2016.

[S9] B. Voisin, J. Salfi, J. Bocquel, R. Rahman, and S. Rogge. Spatially resolved resonant tunneling on single atoms in silicon. *Journal of Physics-Condensed Matter*, 27(15):154203, 2015.

[S10] J. Salfi, B. Voisin, A. Tankasala, J. Bocquel, M. Usman, M. Y. Simmons, L. C. L. Hollenberg, R. Rahman, and S. Rogge. Valley filtering in spatial maps of coupling between silicon donors and quantum dots. *Phys. Rev. X*, 8:031049, Aug 2018.

[S11] Belita Koiller, Xuedong Hu, and S. Das Sarma. Exchange in silicon-based quantum computer architecture. *Phys. Rev. Lett.*, 88:027903, Dec 2001.

[S12] G. Pica, B. W. Lovett, R. N. Bhatt, and S. A. Lyon. Exchange coupling between silicon donors: The crucial role of the central cell and mass anisotropy. *Physical Review B - Condensed Matter and Materials Physics*, 89(23):1–5, 2014.

[S13] Juan P. Dehollain, Juha T. Muhonen, Kuan Y. Tan, Andre Saraiva, David N. Jamieson, Andrew S. Dzurak, and Andrea Morello. Single-Shot Readout and Relaxation of Singlet and Triplet States in Exchange-Coupled P 31 Electron Spins in Silicon. *Physical Review Letters*, 112(23):236801, jun 2014.

[S14] Yu Wang, Archana Tankasala, Lloyd C L Hollenberg, Gerhard Klimeck, Michelle Y Simmons, and Rajib Rahman. Highly tunable exchange in donor qubits in silicon. *Npj Quantum Information*, 2:16008–, April 2016.

[S15] John King Gamble, N. Tobias Jacobson, Erik Nielsen, Andrew D. Baczewski, Jonathan E. Moussa, Inès Montaño, and Richard P. Muller. Multivalley effective mass theory simulation of donors in silicon. *Phys. Rev. B*, 91:235318, Jun 2015.

[S16] M V Klymenko and F Remacle. Electronic states and wavefunctions of diatomic donor molecular ions in silicon: multi-valley envelope function theory. *Journal of Physics: Condensed Matter*, 26(6):065302, jan 2014.

[S17] Shota Nagayama, Austin G Fowler, Dominic Horsman, Simon J Devitt, and Rodney Van Meter. Surface code error correction on a defective lattice. *New Journal of Physics*, 19(2):023050, 2017.

[S18] C. D. Hill, L. C. L. Hollenberg, A. G. Fowler, C. J. Wellard, A. D. Greentree, and H.-S. Goan. Global control and fast solid-state donor electron spin quantum computing. *Phys. Rev. B*, 72:045350, Jul 2005.

[S19] M. J. Testolin, C. D. Hill, C. J. Wellard, and L. C. L. Hollenberg. Robust controlled-not gate in the presence of large fabrication-induced variations of the exchange interaction strength. *Phys. Rev. A*, 76:012302, Jul 2007.

[S20] Jarryd J. Pla, Kuan Y. Tan, Juan P. Dehollain, Wee H. Lim, John J. L. Morton, David N. Jamieson, Andrew S. Dzurak, and Andrea Morello. A single-atom electron spin qubit in silicon. *Nature*, 489:541–, September 2012.
